# Comprehensive voxel-wise, tract-based, and network lesion mapping reveals unique architectures of right and left visuospatial neglect

**DOI:** 10.1007/s00429-023-02702-2

**Published:** 2023-09-11

**Authors:** Margaret Jane Moore, Luke Hearne, Nele Demeyere, Jason B. Mattingley

**Affiliations:** 1 Queensland Brain Institute, University of Queensland, St. Lucia, QLD 4072 Australia; 2 QIMR Berghofer Medical Research Institute, Brisbane, QLD Australia; 3 Department of Experimental Psychology, University of Oxford, Oxford, UK

**Keywords:** Lesion mapping, Stroke, Attention, Visuospatial neglect, Network-level lesion mapping

## Abstract

Visuospatial neglect is a common, post-stroke cognitive impairment which is widely considered to be a disconnection syndrome. However, the patterns of disconnectivity associated with visuospatial neglect remain unclear. Here, we had 480 acute stroke survivors [age = 72.8 (SD = 13.3), 44.3% female, 7.5 days post-stroke (SD = 11.3)] undertake routine clinical imaging and standardised visuospatial neglect testing. The data were used to conduct voxel-wise, tract-level, and network-level lesion-mapping analyses aimed at localising the neural correlates of left and right egocentric (body-centred) and allocentric (object-centred) visuospatial neglect. Only minimal anatomical homogeneity was present between the correlates of right and left egocentric neglect across all analysis types. This finding challenges previous work suggesting that right and left visuospatial neglect are anatomically homologous, and instead suggests that egocentric neglect may involve damage to a shared, but hemispherically asymmetric attention network. By contrast, egocentric and allocentric neglect was associated with disconnectivity in a distinct but overlapping set of network edges, with both deficits related to damage across the dorsal and ventral attention networks. Critically, this finding suggests that the distinction between egocentric and allocentric neglect is unlikely to reflect a simple dichotomy between dorsal versus ventral networks dysfunction, as is commonly asserted. Taken together, the current findings provide a fresh perspective on the neural circuitry involved in regulating visuospatial attention, and provide important clues to understanding the cognitive and perceptual processes involved in this common and debilitating neuropsychological syndrome.

**Supplementary Information:**

The online version contains supplementary material available at 10.1007/s00429-023-02702-2.

## Introduction

Visuospatial neglect is a common neuropsychological condition characterised by consistently lateralised deficits (Parton et al. 2004). Many previous lesion-mapping investigations have aimed to identify the neural correlates of this syndrome, but little consensus has arisen from this work (Moore et al. 2023a, b). This heterogeneity is potentially due to visuospatial neglect being a disconnection syndrome rather than a deficit attributable to a single, spatially contiguous neural correlate (Bartolomeo et al. 2007; Toba et al. 2017). It is, therefore, important to characterise the patterns of disconnectivity associated with visuospatial neglect to provide novel insight into the structural circuitry that regulates spatial attentional processes.

Past research aiming to identify the correlates of visuospatial neglect has most commonly explored the anatomy of left-lateralised, body-centred attentional bias occurring in patients with right-hemisphere lesions (Moore et al. 2023a, b; Parton et al. 2004). However, recent research has demonstrated that visuospatial neglect is highly heterogeneous, with different patients exhibiting attentional biases in different reference frames following a diverse range of both left- and right-hemisphere lesions (Beume et al. 2015; Moore et al. 2021a, b; Ten Brink et al. 2017). Critically, visuospatial neglect can selectively impact an egocentric (body-centred) or allocentric (object-centred) reference frame. Although egocentric and allocentric neglect commonly occur together (Demeyere and Gillebert 2019), research has demonstrated a double dissociation between the two conditions (Bickerton et al. 2011; Demeyere and Gillebert 2019; Ota et al. 2001). Moreover, they appear to be associated with distinct neural correlates (Chechlacz et al. 2010; Kenzie et al. 2015; Moore et al. 2021a, b) and are differentially predictive of patient outcomes (Chechlacz et al. 2010; Moore et al. 2021a, b). For example, Moore et al. (2021a, b) found that the severity of acute allocentric neglect, but not egocentric neglect, was a significant predictor of poor long-term functional outcomes as quantified by the Stroke Impact Scale (Duncan et al. 1999). Similarly, visuospatial neglect impairment has been documented following both left- and right-hemisphere lesions. Recent literature has reported that visuospatial neglect occurs in 40–80% of right-hemisphere and 20–60% of left-hemisphere stroke patients, with estimates varying based on testing method and assessment timepoint (Azouvi et al. 2002; Ringman et al. 2004; Stone et al. 1993; Ten Brink et al. 2017). It is critical that lesion-mapping studies adequately account for this heterogeneity.

Most published studies have focused on reporting correlates of left visuospatial neglect resulting from right-hemisphere lesions (Chechlacz et al. 2012a, b; Molenberghs et al. 2012). Chechlacz et al. (2012a, b) conducted a meta-analysis of right-hemisphere visuospatial neglect lesion-mapping studies. The authors concluded that egocentric neglect is associated with damage to the pre/post-central, supramarginal, and superior temporal gyri, whereas allocentric neglect is associated with the angular and middle temporal gyri (Chechlacz et al. 2012a). Crucially, both egocentric and allocentric neglect were associated with damage to the superior longitudinal, inferior fronto-occipital, and inferior longitudinal fasciculi (Chechlacz et al. 2012a). Despite these overall findings, substantial disagreement is present across studies that have aimed to identify the right-hemisphere correlates of visuospatial neglect. Past research has linked left egocentric neglect to lesions of the right parahippocampal cortex and angular gyrus (Mort et al. 2003) or the white matter tracts connecting these areas (Bird 2006). Karnath et al. (2002) reported that subcortical lesions effecting the right pulvinar, putamen, and caudate nucleus were associated with left egocentric neglect. Visuospatial neglect symptoms have also been identified in patients with exclusively cerebellar lesions (Hildebrandt et al. 2002; Kim et al. 2008; Silveri 2001). Overall, 34 published lesion-mapping studies have associated 72 distinct brain regions with left-lateralised egocentric neglect impairment, with the greatest degree of consensus being that the superior longitudinal fasciculus is associated with left egocentric neglect (14/34 studies)(Moore et al. 2023a, b).

The neuroanatomy of left-hemisphere visuospatial neglect is less well established. Suchan and Karnath (2011) studied the anatomy of right egocentric neglect following lesions to the left hemisphere (*n* = 33), and concluded that this deficit was associated with damage to the middle and superior temporal gyri, insula, and inferior parietal lobule. However, this result has not been replicated by subsequent investigations. Beume et al. (2017) linked right egocentric neglect to the left anterior temporal lobe and left frontal operculum (*n* = 121). Most recently, Moore et al. (2021a, b) found that right egocentric neglect was most closely associated with damage to the left inter/supra calcarine cortex and lingual gyrus (*n* = 446). Notably, only one study to date has reported on the correlates of right-lateralised allocentric neglect (Moore et al. 2021a, b), which was linked to damage of the left anterior limb of the internal capsule and the left external capsule.

To summarise, there is considerable variability in the existing literature on the anatomy of visuospatial neglect. This can partially be accounted for by characterising visuospatial neglect as a disconnection syndrome instead of a deficit arising from damage to any single, spatially contiguous neural region (Bartolomeo et al. 2007; Thiebaut de Schotten et al. 2011). This conceptualisation has important implications for visuospatial neglect lesion-mapping analyses. Traditional univariate lesion-mapping is not generally considered appropriate for determining the neural correlates of disconnection syndromes. This is because the technique averages across affected regions (Bates et al. 2003), and can therefore yield erroneous mean locations rather than identifying the contributions of distinct regions. Similarly, traditional univariate lesion-mapping results are susceptible to spatial misallocations due to the non-random distribution of lesions caused by the brain’s vascular network (Mah et al. 2014). Several alternative analysis techniques have been employed to avoid these potentially confounding issues.

### Network-level lesion-symptom mapping and visuospatial neglect

Network-based and tract-level lesion-symptom mapping techniques aim to take spatially distributed, disconnection-related effects into account when identifying brain–behaviour relationships (Foulon et al. 2018; Fox 2018; Gleichgerrcht et al. 2017). These methodologies consider probability of disconnection of each pre-defined network edge or tract as behavioural predictors rather than the presence/absence of damage on a voxel-by-voxel basis (Foulon et al. 2018). Network symptom mapping approaches have been applied to investigate the anatomy of visuospatial neglect, but previous analyses have only reported on the anatomy left egocentric neglect following lesions to the right hemisphere. Thiebaut de Schotten et al. (2014) conducted both voxel-level and tract-level lesion-mapping in the right hemisphere (*n* = 58). Their voxel-level lesion-mapping linked left egocentric neglect with lesions in the posterior parietal cortex, whereas tract-level lesion-mapping suggested instead that damage to the second branch of the superior longitudinal fasciculus was the best predictor of visuospatial neglect. Subsequent network lesion-mapping investigations have linked visuospatial neglect impairment to the second/third branch of the superior longitudinal fasciculus (Lunven et al. 2013; Machner et al. 2018; Toba et al. 2017), the inferior fronto-occipital fasciculus (Toba et al. 2017), the splenium of the corpus callosum (Lunven et al. 2013), the inferior longitudinal fasciculus (Machner et al. 2018), and the arcuate fasciculus (Machner et al. 2018).

Notably, only one previous study has employed network-level lesion-mapping techniques to elucidate the correlates of both egocentric and allocentric neglect. Saxena et al. (2022) found that the presence of co-occurring egocentric and allocentric neglect was associated with disconnection in tracts linking the right inferior and superior parietal cortex with other brain regions, and in tracts linking the left or right mesial temporal cortex with other regions. Critically, however, Saxena et al. (2022) did not identify any tracts that were significantly associated with the severity of allocentric neglect alone. The authors report that the latter null finding was likely due to a lack of statistical power due to the relatively small number of allocentric patients included in their analysis (*n* = 15) (Saxena et al. 2022). Moreover, they did not control for lesion volume within their disconnectivity analyses. Given that visuospatial neglect is generally associated with larger lesion volumes (Moore et al. 2021a, b; Saxena et al. 2022), it is unclear how much of any given disconnection may be related to general stroke severity rather than to visuospatial neglect-specific impairments.

### Neglect anatomy in the right versus left hemisphere

Finally, there is significant debate over whether visuospatial neglect following left-hemisphere lesions involves damage to anatomical homologues of right-hemisphere areas implicated in visuospatial neglect. Several previous studies have reported similarity between the correlates of egocentric neglect resulting from right and left-hemisphere lesions (Beume et al. 2017; Suchan and Karnath 2011; Toba et al. 2022). However, it is unclear whether this apparent similarity is simply a consequence of the wide variance in right-hemisphere regions that have been associated with visuospatial neglect, reflects the vasculature of underlying stroke, or instead indicates functional similarity across hemispheres. It seems likely that the probability of any given brain region being associated with right-hemisphere visuospatial neglect spuriously increases the chance that a homologous left-hemisphere region will be similarly implicated. This is because a large number of regions spanning almost the entire right hemisphere have been associated with left egocentric neglect, meaning that there is a high probability that the anatomical homologue of any randomly selected right-hemisphere region has previously associated with left egocentric neglect in at least one previous study (Moore et al. 2023a, b). It remains unclear, therefore, whether studies that have tested for neural correlates of visuospatial neglect within a single hemisphere can meaningfully compare their results with those from other studies that included patients with lesions of the opposite hemisphere. It is more appropriate that studies which aim to compare the neural correlates of visuospatial neglect across hemispheres should identify these correlates in *both* hemispheres in a *single* study, and then compare results across hemispheres (Moore et al. 2023a, b).

### Research aims

Our goal in the present study was to apply voxel-wise, tract-level, and network-based lesion-mapping to investigate the neuroanatomy of egocentric and allocentric visuospatial neglect following acute lesions of the left and/or right hemispheres. Critically, we included a large and representative sample of acute stroke survivors (*n* = 480) who were not excluded/included based on lesion location, stroke type, or comorbid cognitive impairments. Our aim was to better delineate the distributed neuroanatomical networks whose damage gives rise to egocentric and allocentric neglect.

## Materials and methods

### Patients

This study is a retrospective analysis of data collected in the Oxford Cognitive Screening (OCS) Programme (Demeyere et al. 2015) and OCS-Care Study (Demeyere et al. 2019) (NHS REC reference 14/LO/0648, 18/SC/0550, 12/WM/00335). These studies include a consecutive cohort of stroke survivors with minimal exclusion criteria. Specifically, all patients who were able to remain alert for 15 min, provide informed consent in line with the declaration of Helsinki, and communicate responses effectively were included. 663 patients had available neuroimaging data displaying visible lesions and had completed visuospatial neglect testing and were therefore considered for inclusion in the present investigation. Patients were not excluded based on stroke territory or location (e.g. cerebellar strokes). Of these patients, 84 were excluded due to evidence of multiple temporally distinct lesions, and 49 were excluded due to incomplete cancellation task data (scores < 5). Finally, 49 patients were excluded due to scan-testing intervals of > 30 days yielding a final sample of 480 patients (mean age = 72.6 (SD = 13.3, range = 26–95), 45.2% female, 7.1% left handed). A subset of this final sample was reported in previous lesion-mapping investigations (Moore et al. 2021a, b; Moore and Demeyere 2022).

The current sample included 178 left hemisphere, 237 right hemisphere, and 65 bilateral stroke patients where bilateral strokes were defined as a single, spatially contiguous lesion crossing the anatomical midline (e.g., a pons haemorrhage). As the aim of this study is to identify the correlates of left and right visuospatial neglect rather than to identify the correlates of visuospatial neglect following left- and right-hemisphere lesions, reported analyses include the full sample of patients regardless of lesion side. Analyses investigating the correlates of visuospatial neglect within left- and right-hemisphere damage subgroups are reported in Supplementary Materials. The majority of included strokes were ischemic (79%), while the remaining 21% were haemorrhages. Scans were collected on average 1.97 days (SD = 5.09, range = 0–53) following stroke. Visuospatial neglect testing was conducted on average 6.14 days (SD = 6.82, range = 0–55) following stroke, resulting in an average test-scan interval of 3.88 days (SD = 6.66, range = 0–30).

## Behavioural data

**Fig. 1:**
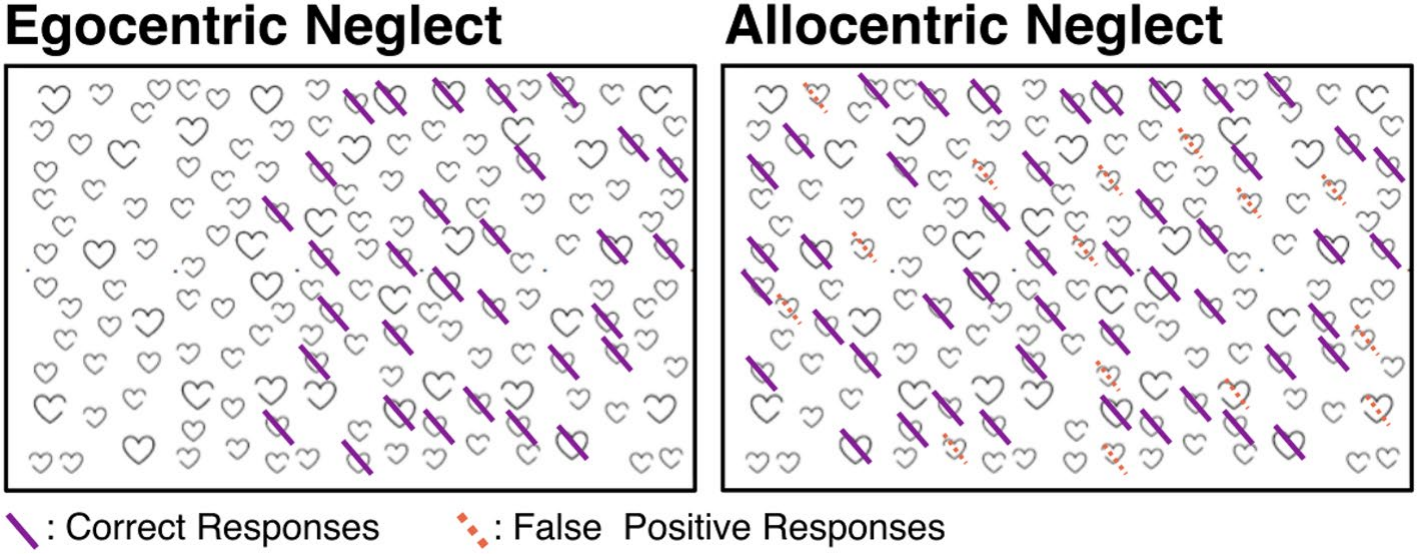
A visualisation of egocentric/allocentric neglect as quantified by the Oxford Cognitive Screen’s Hearts Cancellation Task. Patients exhibiting egocentric neglect impairment fail to report targets on one side of the page. Conversely, patients with allocentric neglect report consistently lateralised false-positive errors

Visuospatial neglect impairment was quantified using the OCS Cancellation Task, administered by trained researchers or occupational therapists. This task is highly sensitive to visuospatial neglect within both egocentric and allocentric reference frames (Demeyere et al. 2015) and is recommended as a first-line screen for visuospatial neglect impairment in clinical environments (Moore et al. 2022). In this task, participants are presented with a search matrix of heart line drawings distributed across a full sheet of paper (Fig. 1), each of which is either complete or incomplete. Incomplete hearts have a small gap in their left side or right side with equal probability. Participants are instructed to mark all complete hearts while ignoring the distractor stimuli (incomplete hearts) (Demeyere et al. 2015). Participants are allocated 3 min to complete this test and are given two practice trials prior to starting. To ensure that only participants who were able to reliably complete the cancellation task were included, data from participants who failed to exhibit adequate task comprehension, failed to complete the task due to fatigue, or were not able to identify at least five correct targets were excluded. According to OCS scoring guidelines, egocentric neglect severity is scored by dividing the search array into five equal columns and subtracting the number of complete hearts reported in the two left-most columns from the number reported in the two right-most columns. Importantly, targets identified in the central column are not included in egocentric asymmetry calculations (Demeyere et al. 2015).

Allocentric neglect severity is scored by subtracting the number of reported right-gap false-positive hearts from the number of left-gap false-positive responses (Demeyere et al. 2015). This comparison between the number of left- and right-gap false positives prevents potential executive function or memory deficits masquerading as allocentric neglect, as the former behaviours would not result in consistently lateralised false-positive errors (Demeyere et al. 2015). In line with the standard impairment thresholds, egocentric scores of > 2 or < -2 represent significant impairment, whereas allocentric scores of > 1 or < -1 represent significant impairment (Demeyere et al. 2015).

For participants exhibiting significant visuospatial neglect impairment according to normative data cut-offs (Demeyere et al. 2015), the standard OCS scoring metrics were transformed to facilitate more fine-grained quantification. These alternative scoring metrics were employed, because the same OCS asymmetry scores can arise from very different behavioural patterns (Demeyere et al. 2015; Moore et al. 2021a, b), and because past work has suggested that “centre of cancellation” metrics offer a more precise method for quantifying the severity of visuospatial neglect impairment (Rorden and Karnath 2010).

The severity of egocentric neglect was quantified by calculating the centre of cancellation (Rorden and Karnath 2010) which summarises the location of the “centre of mass” of identified targets. The severity of allocentric neglect was quantified by dividing the number of consistently lateralised false-positive responses by the total number of correctly identified targets to control for the percentage of the overall search matrix explored by each patient (Moore et al. 2021a, b). This metric quantifies the severity of allocentric neglect independently of the egocentric location of the identified stimuli. Both these metrics can be applied in both left- and right-hemisphere stroke patients with negative scores representing right visuospatial neglect and positive scores representing left visuospatial neglect.

## Neuroimaging data

**Fig. 2:**
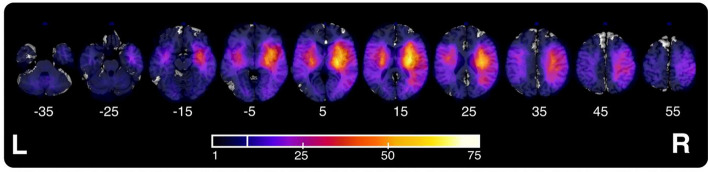
An illustration of the lesion coverage for the full sample of 480 patients. Colour visualises the number of lesions overlapping at each voxel. Only regions impacted by at least ten lesions (marked on colour key) are included in voxel-wise lesion-mapping analyses. MNI z-slice number is reported below each slice

**Fig. 3:**
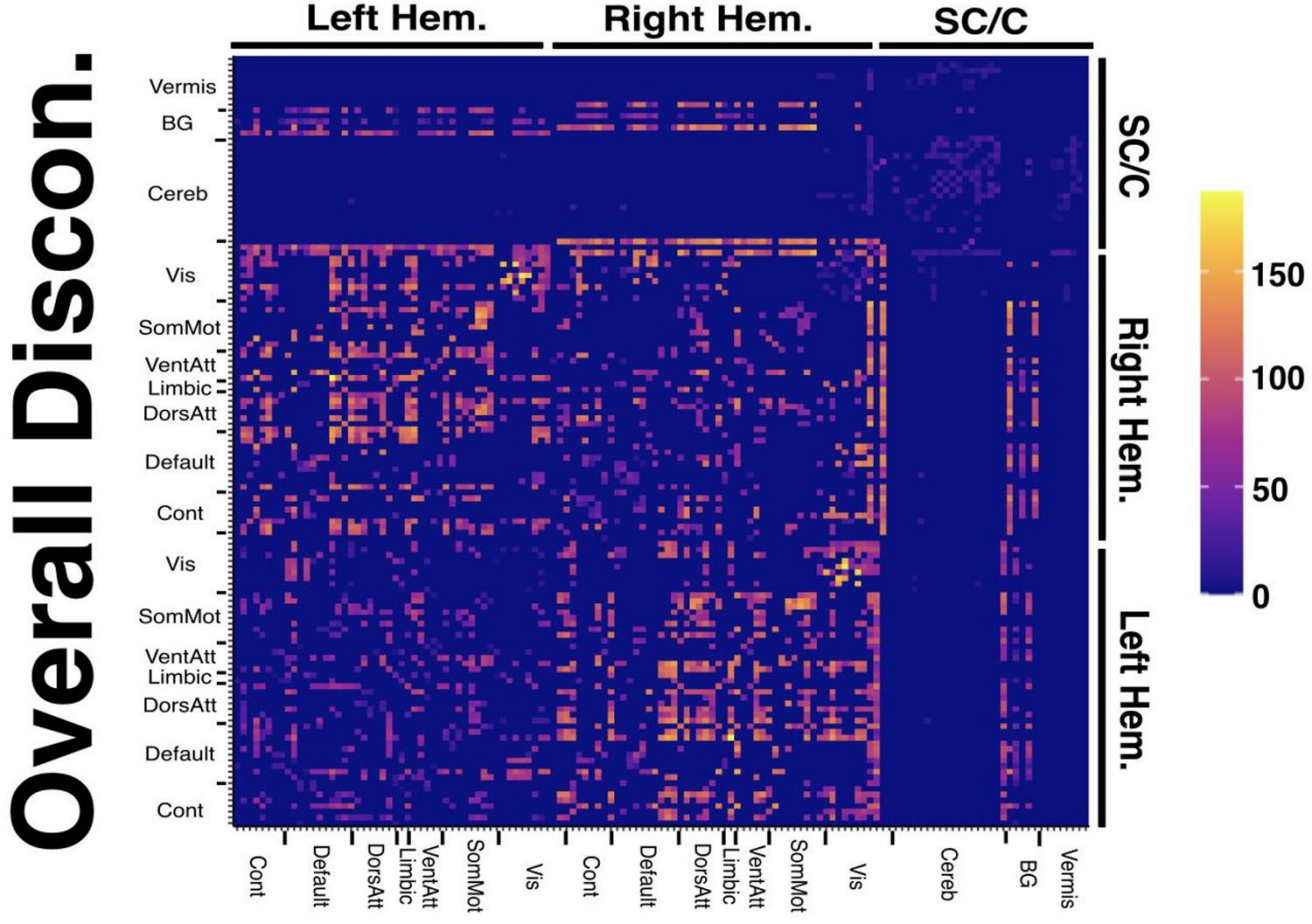
A disconnection matrix illustrating the number of included patients with at least 50% disconnection at each considered network edge. Cell colour represents number of patients. X and Y atlases are arranged according to the parcels and network subdivisions reported in the Schaefer–Yeo Atlas (7 networks, 100 nodes). *SC*  subcortical, *C/Cereb*  cerebellar, *BG* basal ganglia, *Vis* visual network, *SomMot* somatic-motor network, *VentAtt* ventral attention network, *Limbic* limbic network, *DorsAtt* dorsal-attention network, *Default* default network, *Cont* control network. Disconnection matrices for each considered patient sub-group are provided in Supplementary Fig. 3

**Fig. 4:**
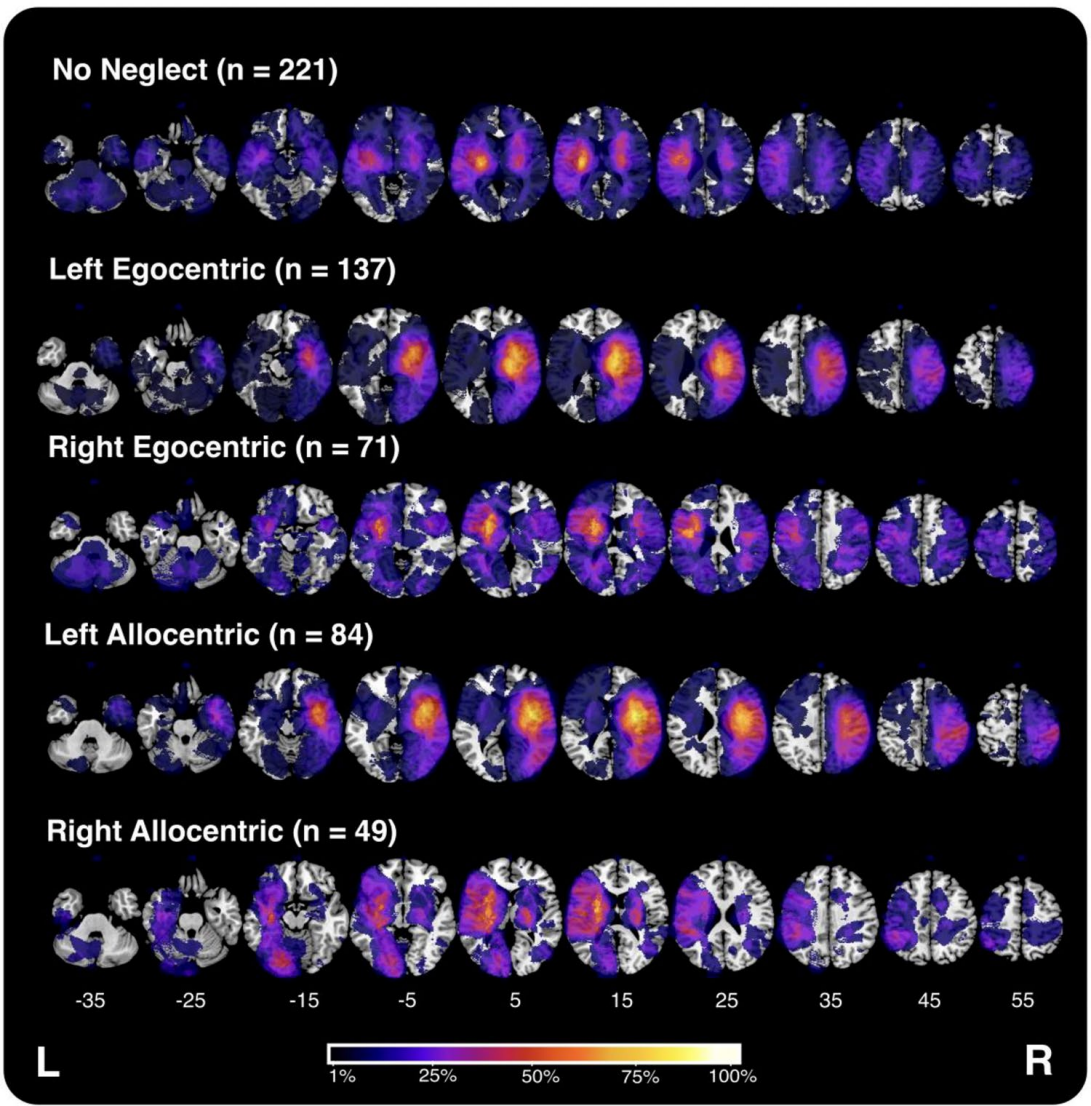
Visualisation of lesion overlays for each of the visuospatial neglect impairment groups investigated in this experiment. As each overlay represents data from a variable number of patients, the colour scale depicts the percentage of included individuals with damage in each voxel relative to the maximum overlap within each sub-group

Binarized lesion masks were created for each patient using routinely available clinical neuroimaging (400 CT, 70 T2, 7 FLAIR, and 3 T1). We have recently verified that such routine clinical imaging data are of sufficient quality to reveal underlying neural correlates of behavioural deficits (Moore and Demeyere 2022) and that lesion-mapping investigations employing CT-derived lesion masks demonstrate good agreement with those performed using MR-derived lesions (Moore et al. 2023a, b). The lesion masks were derived and processed in line with a standardised protocol (Moore 2022). Specifically, all lesions were manually delineated on native-space scans by trained experts and smoothed at 5 mm full width at half maximum in the z-direction using MRIcron (Rorden 2007). These native-space masks and scans were then reoriented to the anterior commissure to reduce transformation degrees of freedom and improve normalisation accuracy (Moore 2022) and warped into 1 × 1 × 1 MNI space using Statistical Parametric Mapping (Ashburner et al. 2016) and standard, age-matched templates (Clinical Toolbox, Rorden et al. 2012). The normalised versions of all scans and lesions were visually inspected to ensure accuracy. Figures 2, 3 present the group-level lesion overlay used in this study, while Fig. 4 illustrates the lesion overlap within each relevant impairment sub-group. Figure 3 illustrates the group-level disconnection profile of this sample.

## Voxel-wise lesion mapping

In a first step, traditional univariate lesion-mapping analyses were conducted to identify voxels that, when damaged, are associated with visuospatial neglect impairment. These analyses were included to ensure the voxel-level correlates of visuospatial neglect within the used sample align with what has been reported by previous analyses. Specifically, four theory-blind, voxel-wise lesion-mapping analyses were conducted to identify voxels associated with left egocentric, left allocentric, right egocentric, and right allocentric neglect independently, using LESYMAP (Pustina et al. 2018). These analyses only considered voxels which were damaged in > 10 patients to prevent outlier voxels from creating spatial bias in the resultant statistical maps (Sperber and Karnath 2017). As there is no standard approach to determining the exact minimum overlap threshold to employ in lesion mapping studies, this comparatively conservative approach was adopted to minimise the risk of voxel-wise results being biased by single outlier data points (Moore et al. 2023a, b; Sperber and Karnath 2017). Each analysis included the full sample of patients, meaning that voxel-wise statistical power remained constant across all conducted voxel-wise analyses. These analyses controlled for lesion volume by employing “direct total lesion volume control” (Pustina 2019; Zhang et al. 2014), which places greater weight on the impact of smaller lesions. In this method, binarised lesion mask voxels are divided by the square root of lesion volume prior to analysis (Zhang et al. 2014). The results of the lesion-mapping analyses were corrected for multiple comparisons using strict, conservative Bonferroni corrections (voxels tested = 691,776, corrected alpha = 7.23 × 10^–8^). These highly conservative statistical corrections were used to exploit our large sample to prioritise result specificity over sensitivity (Sperber and Karnath 2017). Specifically, this study aims to localise “core” lesion sites which are strongly associated with the behaviour or interest rather than peripheral, less strongly associated regions.

Across all conducted lesion mapping analyses, patients with significant impairment in the condition of interest were assigned the relevant visuospatial neglect severity score, whereas scores for all other patients were constrained to zero. For example, in analyses investigating left egocentric neglect, all patients with right egocentric neglect were assigned egocentric severity scores of zero. This approach ensured that each analysis identified correlates of the behaviour of interest without interference from the opposite neglect lateralization. This approach was used in several previous studies whose aim was to quantify the severity of visuospatial neglect as captured by the OCS (Moore et al. 2021a, b). The approach also facilitates identification of areas associated with both left and right visuospatial neglect impairment.

## Tract-level lesion mapping

Tract-level lesion-mapping analyses were conducted to evaluate whether the severity of visuospatial neglect was predicted by damage to specific white matter tracts, as defined by a standard, normative atlas. Lesion Quantification Toolkit (Griffis et al. 2021) was employed to calculate the percentage of streamlines disconnected within each of the 70 tracts defined in the normative HCP-842 Atlas (Yeh et al. 2018). This atlas was derived by compiling high-resolution diffusion tractography from 842 participants, representing 550,000 white matter fascicles. These fascicles were categorised under anatomical labels by a team of expert neuroanatomists (Yeh et al. 2018). Notably, this atlas does not contain defined ROIs for all potential tract subdivisions (e.g., separate branches of the superior longitudinal fasciculus). Full details of each considered ROI are available in Yeh et al. (2018).

For each of these normative tracts, a regression analysis was conducted to determine whether percent disconnection was related to visuospatial neglect severity. This analysis structure is identical to the analyses conducted in voxel-wise lesion-mapping comparisons, but considers tract-level rather than voxel-level damage statistics as predictor variables (Bates et al. 2003; Thiebaut de Schotten et al. 2014). Each tract-level analysis included the full sample of patients. These analyses included lesion volume as a covariate and were corrected for multiple comparisons using highly conservative Bonferroni corrections (alpha = 0.0007). In cases where no tracts survived this conservative correction, a 5% False Discovery Rate correction for multiple comparisons was employed. This exploratory approach was adopted to minimise false-negative results. These can arise when employing strict Bonferroni corrections, which are widely regarded as being too conservative for lesion-mapping analyses (Mirman et al. 2018). Past lesion mapping studies have used similar correction threshold adjustments (Machner et al. 2018; Moore et al. 2023a, b). Four separate tract-based analyses were performed to identify tracts associated with left egocentric, left allocentric, right egocentric, and right allocentric neglect independently. Tract-level analyses were performed using original scripts written in R (available at https://osf.io/qwd8k/).

## Network-level lesion-symptom mapping

Binarized lesion masks were overlayed onto the Schaefer–Yeo Atlas Parcellation (100 parcels, 7 Networks) (Schaefer et al. 2018) to estimate parcel-wise disconnection severities by calculating the number of HCP-842 streamlines which bilaterally terminate within each pair of included grey matter parcels (Griffis et al. 2021). Additional 35 subcortical and cerebellar parcels derived from the AAL (Tzourio-Mazoyer et al. 2002) and Harvard–Oxford Subcortical Atlas, respectively, were also included. This process yields disconnection matrices in which the value in each cell represents the percentage of disconnected streamlines (edges) connecting each of the defined grey matter parcels (nodes) per patient (135 nodes, 18,225 edges) (Griffis et al. 2021). For each network edge, a regression was conducted to compare percent disconnection to visuospatial neglect severity. Each regression employed the full sample of patients, controlled for lesion volume, and only edges damaged in > 10 patients were analysed (2,708 edges included, alpha = 1.85 × 10^–5^). Four separate network-level analyses were performed to identify tracts associated with left egocentric, left allocentric, right egocentric, and right allocentric neglect independently. This approach is similar to network-level lesion-mapping methodologies employed in the previous studies (Gleichgerrcht et al. 2017; Saxena et al. 2022). These network-level analyses were performed using original scripts written in R (available at https://osf.io/qwd8k/).

## Secondary analyses

There is debate within the visuospatial neglect lesion-mapping literature as to whether the correlates of visuospatial neglect impairment are homologous across hemispheres (Beume et al. 2017; Moore et al. 2021a, b; Suchan and Karnath 2011). To address this issue, results of our voxel-wise, tract-level, and network-level lesion-mapping results were analysed to evaluate the degree of overlap between the correlates of left- and right-lateralised visuospatial neglect. At each analysis level, the left-hemisphere statistical maps (significant voxels, tracts, or edges) summarising areas associated with visuospatial neglect deficits were inverted, so that they could be overlaid on the corresponding homologues in the right hemisphere. The goal of this approach was to identify areas of overlap in the correlates of visuospatial neglect following lesions of the right and left hemispheres. For voxel-level analyses, Dice similarity coefficients were calculated to quantify the degree of overlap between results clusters. The Dice similarity coefficient is a standard metric used to quantify the degree of similarity between voxel maps. This metric is calculated by dividing the number of overlapping voxels (e.g., the number of homologous voxels between right and left neglect) by the average number of voxels included in the individual masks (e.g., the number of significant voxels associated with right and left neglect). In line with standard interpretation thresholds, Dice similarity coefficients of > 0.8 represent excellent overlap, > 0.6 represent substantial agreement, > 0.4 represent moderate agreement, > 0.2 represent slight agreement, and < 0.2 represents poor agreement (Landis and Koch 1977).

## Results

### Behavioural descriptive

Overall, 259/480 (53.95%) of the included participants exhibited visuospatial neglect according to normative impairment thresholds (Table 1). This sample included 137 cases of left egocentric, 71 cases of right egocentric, 84 cases of left allocentric, and 49 cases of right allocentric neglect, as defined by standard neglect scoring procedures. For patients exhibiting egocentric neglect, 76.1% of right-hemisphere patients exhibited left-lateralised impairment and 55.3% of left-hemisphere patients exhibited right-lateralised impairment. For patients exhibiting significant allocentric neglect, 78.4% of right-hemisphere patients demonstrated left-lateralised deficits and 63.9% of left-hemisphere patients exhibited right-lateralised impairments. For bilateral patients with neglect, 66.7% of egocentric and 56.5% of allocentric cases were left lateralised (see Table 1). For those patients with neglect, egocentric and allocentric neglect severity scores were significantly correlated (Kendall’s tau = 0.163, *z* = 3.75, *p* < 0.001).

**Table 1:** Demographics, stroke lesion descriptives, and visuospatial neglect assessment performance summary for all included patients

	Demographics					Lesion descriptives				Neglect testing		
	N	%F	Age	Education	%L Hand	L	R	B	Volume	CoC	AlloProp	Total
No neglect	221	40.00%	70.2 (15.0)	12.2 (2.9)	6.12%	108	86	27	2.89 (4.21)	0 (0.07)	0 (0.02)	41.8 (9.44)
Left egocentric	137	49.20%	73.9 (11.3)	12.2 (3.8)	5.45%	21	96	20	7.07 (8.17)	0.83 (0.68)	0.17 (0.35)	24.5 (13.4)
Left allocentric	84	45.00%	73.5 (12.4)	13.2 (4.4)	6.25%	13	58	13	8.28(9.73)	0.74 (0.81)	0.37 (0.34)	23.5 (14.2)
Right egocentric	71	49.20%	75.6 (10.8)	11.8 (2.9)	6.45%	26	30	10	3.71 (5.82)	− 0.43 (0.47)	− 0.03 (0.09)	33.6 (11.4)
Right allocentric	49	53.30%	75.2 (11.2)	11.1 (2.11)	8.69%	23	16	10	4.33 (7.15)	− 0.04 (0.57)	− 0.16 (0.17)	33.2 (13.8)

Notably, some patients exhibited more than one type of visuospatial neglect (e.g., left egocentric and allocentric impairment). Data are presented as means and standard deviations (in parentheses). Raw egocentric scores of ≥ 3 (mean CoC = 0.129) or ≤ −3 (mean Coc = −0.123) indicate significant visuospatial neglect. Raw allocentric scores of > 1 (mean proportion score = 0.091) or < −1 (mean proportion score = −0.063) indicate significant impairment

*R* Right, *L* Left, *B* Bilateral, *F* Female, *CoC* Center of Cancellation, *AlloProp* Allocentric Proportion, *Total* OCS Cancellation Task total score

Patients with visuospatial neglect impairment had significantly larger lesions than patients without visuospatial neglect [*t*(411.5) = 5.39, *p* < 0.001, 95% CI 1.91–4.09 (cm^3^)]. Patients with left-lateralised visuospatial neglect had larger lesions than patients with right visuospatial neglect (*t*(407.7) = 2.39, *p* = 0.0175, CI 0.26–2.72(cm^3^)).

However, lesion size was not significantly different between patients with different visuospatial neglect subtypes (*F*(2,256) = 2.813, *p* = 0.0619). Overall, 208 patients had significant egocentric neglect and 133 patients had significant allocentric neglect, with 82 patients exhibiting both egocentric and allocentric impairment. Lesion overlays for patients with both egocentric and allocentric neglect are provided in the Supplementary Materials.

## Voxel-wise lesion-mapping results

**Fig. 5:**
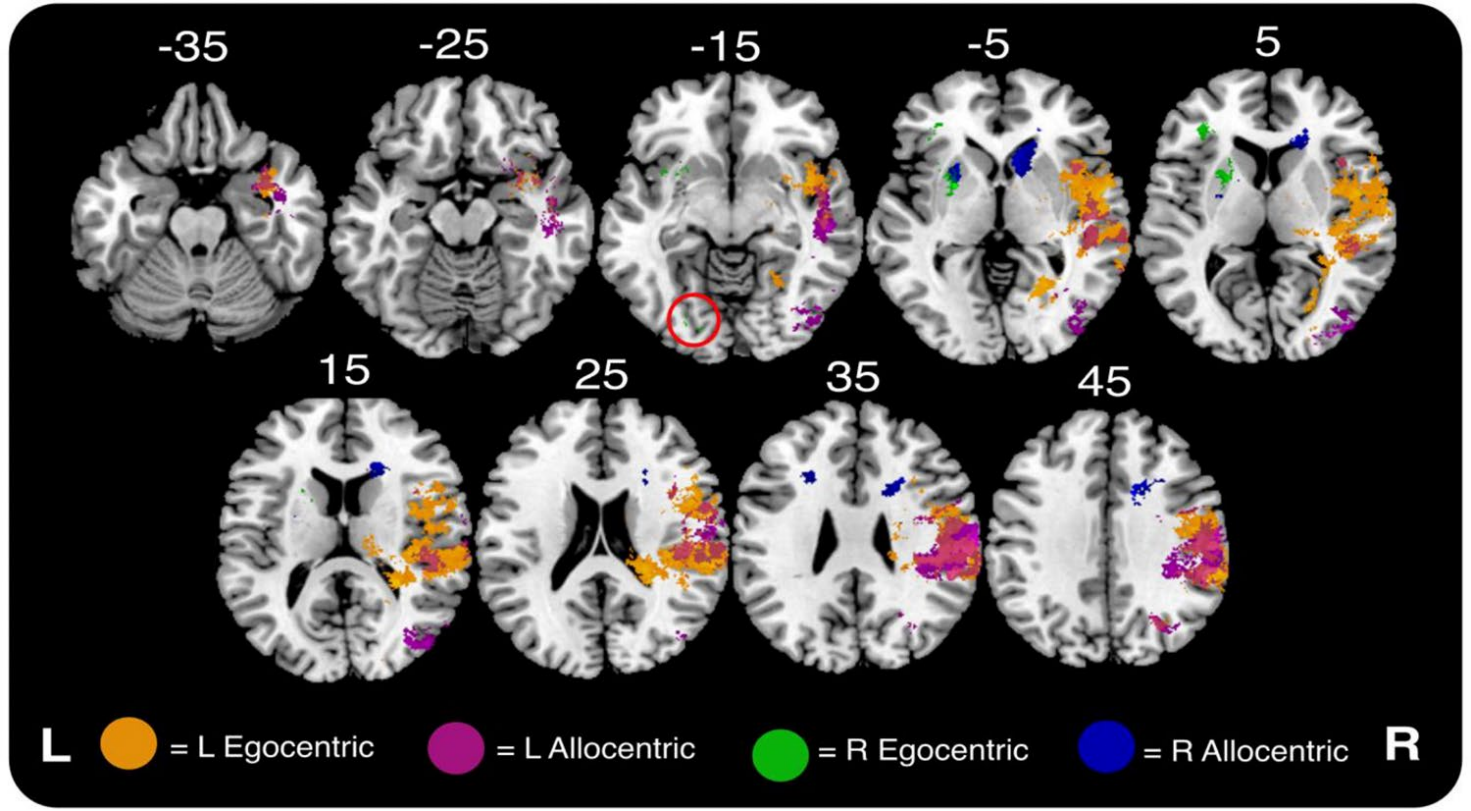
Visualisation of significant voxels identified in univariate lesion-mapping analyses of left and right egocentric and allocentric neglect. All visualised voxels survived highly conservative Bonferroni corrections for multiple comparisons. The posterior component associated with right egocentric neglect is highlighted by a red circle. MNI z coordinates −35–45 are visualised

Left egocentric neglect was significantly associated with a large cluster of voxels [*n* = 40,209 (total volume = 40.2 cm^3^)] centred within the posterior division of the right superior temporal gyrus (peak *z*-score = 10.51, MNI 51 -15 -9). These significant voxels were distributed throughout the posterior parietal cortex, temporo-parietal junction, and opercular cortices (Fig. 5, Table 2). Conversely, right egocentric neglect was significantly associated with 1,739 voxels (total volume = 1.73 cm^3^) with the highest z-score falling near the boundary of the left amygdala and inferior putamen (MNI -25 2 -11). These results were organised in three distinct clusters located within the left occipital fusiform gyrus, frontal orbital cortex, and the putamen and surrounding white matter.

**Table 2:** Anatomical statistics for each of the conducted univariate lesion-mapping analyses

ROI name	Egocentric		Allocentric	
	Left (%)	Right (%)	Left (%)	Right (%)
Acoustic radiation	17.64		0.10	
Angular gyrus	6.34		4.46	
Anterior commissure	2.16	0.01	1.50	
Arcuate fasciculus	4.35		4.35	0.06/0.01*
Central opercular cortex	48.45		3.87	
Cingulum				2.28
Corpus callosum	3.05	0.08	2.98	0.65
Cortico spinal tract	6.64		2.13	0.04/0.46*
Cortico striatal pathway	2.57	0.55	1.45	0.31/1.98*
Corticothalamic pathway	7.00	0.34	5.69	0.25/1.28*
Extreme capsule	13.05	0.06	2.34	
Frontal aslant tract	7.52		2.54	1.01/1.53*
Frontal operculum cortex	13.24	0.37	7.22	0.02
Frontopontine tract	0.76	0.89	0.38	1.00/2.53*
Heschl’s gyrus	72.77		12.39	
Inferior frontal gyrus (pars opercularis)	14.96		1.49	
Inferior frontal gyrus (pars triangularis)	0.89	0.77		
Inferior fronto-occipital fasciculus	3.33	0.44	0.10	0.01
Inferior longitudinal fasciculus	0.80	0.01	7.07	
Insular cortex	25.45	1.00	3.60	0.02
Intracalcarine cortex	7.94			
Lateral occipital cortex (inferior division)	0.09		10.15	
Lateral occipital cortex (superior division)	2.47		3.95	
Lingual gyrus	4.40	0.02		
Middle longitudinal fasciculus	41.60		8.22	
Middle temporal gyrus (posterior division)	4.26		10.99	
Middle temporal gyrus (temporo-occipital part)	3.11		1.12	
Occipitopontine tract	4.90		1.56	
Optic radiation	4.11			
Parietal operculum cortex	79.61		60.83	
Planum polare	41.92		21.30	
Planum temporale	64.57		19.85	
Postcentral gyrus	10.23		26.25	
Precentral gyrus	10.73		5.06	
Superior longitudinal fasciculus	9.79	0.08	7.30	0.52/0.40*
Superior parietal lobule	0.02		4.12	
Superior temporal gyrus (anterior division)	4.60		4.45	
Superior temporal gyrus (posterior division)	32.45		22.35	
Supramarginal gyrus (anterior division)	46.43		66.80	
Supramarginal gyrus (posterior division)	29.94		22.18	
Temporal pole	2.68	0.01	1.84	
Temporopontine tract	8.84		0.14	
U fibre	8.49	0.01	9.43	0.14/0.43*
Uncinate fasciculus	11.70	0.55	10.71	0.04
Vertical occipital fasciculus	0.19		4.67	

Anatomy is reported relative to the Harvard Oxford Cortical Atlas and HCP-842 Atlas. Values represent the percent of each defined region of interest found to be significant in each analysis. In cases where more than ten areas were associated with an outcome variable, unique regions with fractions < 1% are not reported. All reported fractions refer to contralesional deficits (e.g., left visuospatial neglect associated with right-hemisphere damage), with the exception of starred values which represent ipsilesional associations

Left allocentric neglect was significantly associated with damage to 40,209 voxels (total volume = 40.2 cm^3^) with the maximum *z*-value centred in the right supramarginal gyrus (anterior division) (peak *z*-value = 10.70, MNI 63 -20 34). Significant voxels were distributed throughout the left temporo-parietal junction, posterior parietal cortex, and lateral occipital cortex (Fig. 5).

Conversely, right allocentric neglect was associated with 4,432 significant voxels (total volume = 4.4 cm^3^) located in both the left and right-hemisphere internal capsule (anterior division) and corona radiata (anterior division) (peak *z*-score = 12.18, MNI 15 8 26). Specifically, these voxels were organised into clusters centred within the left superior corona radiata, right anterior corona radiata, the intersection of the right caudate nucleus, putamen, and pallidum, and the intersection of the left-hemisphere putamen and pallidum. Little overlap was present within the cross-hemisphere anatomical homologues of the voxels associated with visuospatial neglect in the left and right hemispheres. Only 9 (0.02%, volume = 0.009 cm^3^) voxels associated with left egocentric neglect overlapped with voxels in the opposite hemisphere associated with right egocentric neglect. This overlap yields a Dice score of 0.00043, representing very poor overlap between the two compared results masks. There was no overlap between the voxels associated with right allocentric neglect and left-hemisphere homologues of regions associated with left allocentric neglect. This comparison yielded a Dice score of 0, representing no overlap between the two compared results masks.

## Tract-based lesion-mapping results

**Fig. 6:**
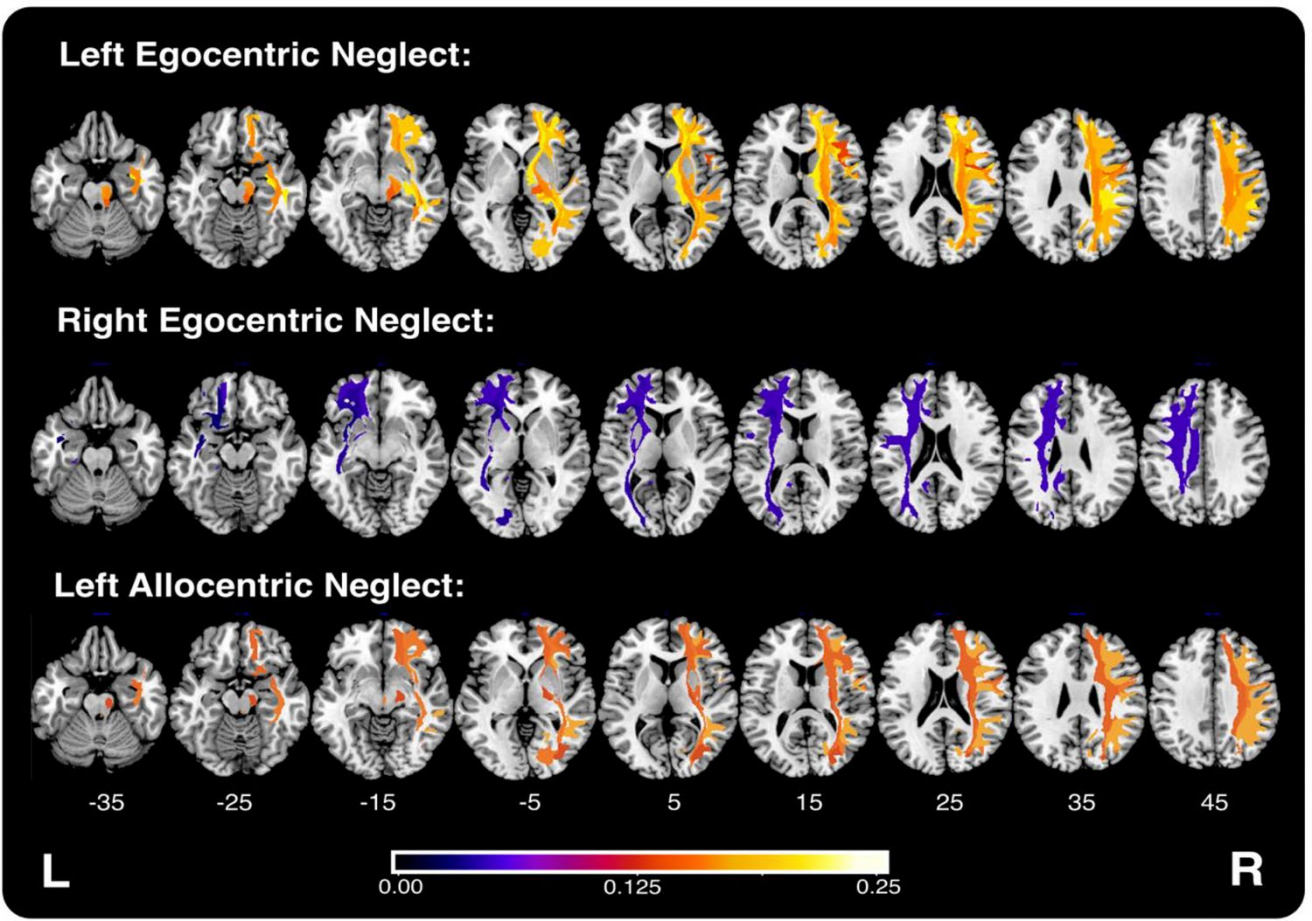
Visualisation of significant tracts identified in lesion-mapping analyses of right and left allocentric and egocentric neglect. Tract colour indicates adjusted *R*^2^ value of the regression analyses predicting visuospatial neglect impairment from percent disconnection (controlling for lesion volume). MNI z coordinates −35–45 are visualised

Left egocentric neglect was associated with disconnection in a large number (*n* = 21) of right-hemisphere tracts spanning parietal, frontal, temporal, and occipital regions (Fig. 6, Table 3). The greatest proportion of variance in left egocentric neglect severity was accounted for by damage to the right corticothalamic pathway (adjusted R^2^ = 0.218) and the inferior fronto-occipital fasciculus (adjusted *R*^2^ = 0.203) (Table 3). Conversely, right egocentric neglect impairment was associated with disconnection within four left-hemisphere tracts including the cingulum (adjusted *R*^2^ = 0.044), corticostriatal pathway (adjusted *R*^2^ = 0.039), inferior fronto-occipital fasciculus (adjusted *R*^2^ = 0.027), and the uncinate fasciculus (adjusted *R*^2^ = 0.021). Left allocentric neglect impairment was associated with disconnection in 11 right-hemisphere tracts including the U-fibres (adjusted *R*^2^ = 0.180), uncinate fasciculus (adjusted *R*^2^ = 0.152), and inferior longitudinal fasciculus (adjusted *R*^2^ = 0.151). Right allocentric impairment was not significantly associated with disconnection in any of the considered tracts, even when less conservative FDR corrections were applied.

**Table 3:** Results from each of the tract-level lesion-mapping analyses

Tract name	Egocentric		Allocentric
	Left	Right	Left
Arcuate fasciculus	0.18		0.15
Acoustic radiation	0.19		
Corticospinal tract	0.19		0.13
Corticostriatal pathway	0.18	0.04	0.14
Corticothalamic pathway	0.22		
Cingulum		0.04	
Extreme capsule	0.19		0.14
Frontal aslant tract	0.13		
Frontopontine tract	0.17		
Fornix	0.17		
Inferior fronto-occipital fasciculus	0.20	0.03	0.15
Inferior longitudinal fasciculus	0.16		0.15
Medial lemniscus	0.15		
Middle longitudinal fasciculus	0.17		0.15
Occipitopontine tract	0.17		
Optic radiation	0.19		
Parietopontine tract	0.16		
Superior longitudinal fasciculus	0.16		0.15
Spino thalamic tract	0.14		
Temporopontine tract	0.16		
Uncinate fasciculus	0.16	0.02	0.14
U fibre	0.19		0.18
Ventral occipital fasciculus			0.13

Anatomy is reported relative to the HCP-842 Atlas. Values represent the adjusted *R*^2^ value of each significant regression analysis. All reported fractions refer to contralesional deficits (e.g., left visuospatial neglect associated with right-hemisphere damage)

Overall, 3/4 of the anatomical homologues of tracts associated with right egocentric neglect were also implicated in left egocentric neglect. Specifically, right egocentric neglect was associated with damage to the corticostriatal pathway, inferior fronto-occipital fasciculus, and uncinate fasciculus (also associated with left egocentric neglect) as well as damage to the cingulum (not associated with left egocentric neglect). However, the tracts which were most strongly associated with left egocentric impairment (e.g., the corticothalamic pathway) were not predictive of right egocentric neglect, and the tract which explained most variance in the severity of right egocentric neglect (cingulum) was not significantly associated with left egocentric neglect.

## Network-based lesion mapping

**Fig. 7:**
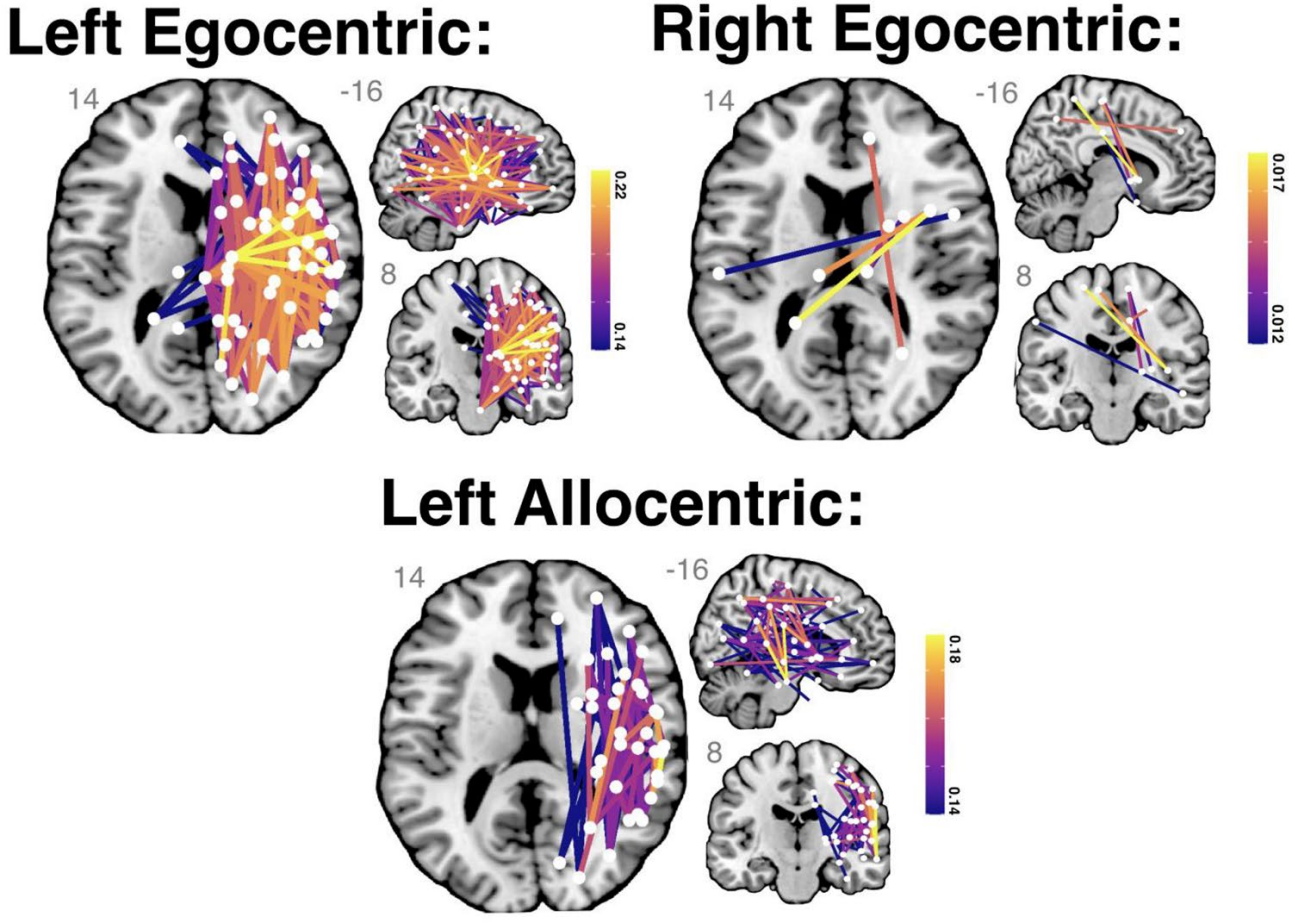
Schaefer–Yeo Atlas network edges significantly associated with each considered visuospatial neglect impairment. Nodes are plotted as white dots and edge colour represents adjusted *R*^2^ value for each base regression analysis. On each slice, node locations are collapsed into two dimensions and plotted in order of ascending *R*^2^ value (highest *R*^2^ values on top). MNI coordinates of each visualised slice are reported in grey. Names, MNI coordinates, and statistics for each significant node are openly available (https://osf.io/qwd8k/)

Left egocentric neglect was significantly associated with the percent disconnection in 232 edges spanning the right hemisphere (Fig. 7). These included edges within the right visual, somatic-motor, ventral attention, dorsal attention, limbic, default, and control networks as defined by the Schaefer–Yeo Atlas (Schaefer et al. 2018). The edges explaining the greatest proportion of variance in left egocentric neglect severity (adjusted *R*^2^ > 0.22) mainly involved connections between the right caudate nucleus and grey matter parcels within the ventral attention network (temporo-occipital part) and dorsal attentional network (precentral ventral parts), somatic-motor network (division 3), and ventral attention network (temporo-occipital part 3).

Conversely, right egocentric neglect was linked to disconnection in six edges connecting the right- and left-hemisphere dorsal attention, ventral attention, default, and somatic-motor networks (FDR-corrected). Specifically, the edges connecting the right-hemisphere ventral attention network (frontal operculum insula division, MNI 40 9 2) with the left-hemisphere dorsal-attention network (posterior division 6, MNI-22-50 67) and the left somatic-motor network division 6 (MNI -11 -25 65) were significant. The network edges connecting the right somatic-motor network division 8 (MNI 11 -23 66) and right thalamus and the right lenticular nucleus were also significant. Finally, the edge connecting the right default network (temporal division 2, MNI -51 7 -16) and left dorsal attentional network (posterior division 2, MNI -49 -23 43) and the edge connecting the right default network dorsal/medial prefrontal cortex division 2 (MNI 12 47 14) and the right dorsal-attention network posterior division 4 (MNI 49 -23 43) were significant. This result cannot be fully accounted for by differences in power between interhemispheric and intrahemispheric connections as 460 tested network edges were impacted in more patients than the average amongst significant interhemispheric edges, but only 5 of these were found to be significant. Similarly, the number of lesions impacting the significant interhemispheric edges was not significantly different from the mean of the tested sample (*t*(2.006) = 2.076, *p* = 0.172, CI: −40.7–117.4).

None of the left-hemisphere network edges that were anatomically homologous to the edges associated with the severity of left egocentric neglect were associated with right egocentric neglect. However, 5/6 edges associated with right egocentric neglect severity were also associated with the severity of left egocentric neglect. Specifically, the edge connecting the left dorsal attentional network (posterior division 2) and the right default network (temporal division 2) was associated with right but not left egocentric neglect. All other significant edges were associated with both right and left egocentric neglect.

The severity of left allocentric neglect impairment was significantly associated with percent disconnection within 73 network edges, spanning right-hemisphere cortical and subcortical areas (Fig. 7). The greatest proportion of variance in left allocentric scores (adjusted R^2^ > 0.18) was accounted for by damage to the edge connecting the right-hemisphere ventral attention network [temporo-occipital part 1 (MNI 58 -41 14)] with the right default network (temporal division 1, MNI 61 -22 -17). Right allocentric neglect severity was not significantly associated with disconnection within any of the considered network edges (maximum *R*^2^ = 0.002, FDR-corrected *p *value = 0.369). The disconnection correlates of left allocentric neglect largely overlapped with the edges significantly associated with left egocentric neglect impairment (70/73 edges shared). However, three edges were significantly associated with the severity of allocentric but not egocentric impairment: an edge connecting the frontal eye fields (dorsal-attention network, MNI 28 -1 50) and the lateral prefrontal cortex (control network division 4, MNI 43 17 46), an edge connecting the default network dorsal/medial prefrontal cortex (division 3, MNI 26 25 50) and the dorsal attentional network posterior division 3 (MNI 39 -44 50), and an edge connecting the somatic-motor network division 6 (MNI 41 -21 61) and 7 (MNI 30 -36 65).

## Discussion

Our goal was to quantify the voxel-wise, tract-level, and network-level anatomy of egocentric and allocentric visuospatial neglect in a representative sample of stroke patients. Importantly, we employed highly conservative methods and corrections in a large sample to provide novel insight into the “core” correlates underlying common subtypes of visuospatial neglect. Our voxel-wise results agree well with the previous literature, linking left egocentric and allocentric neglect to partially overlapping right temporo-parietal regions (Chechlacz et al. 2012a; Molenberghs et al. 2012). Right egocentric neglect was significantly associated with damage to the left occipital fusiform gyrus, whereas right allocentric neglect was related to damage to voxels in both the white matter of the left and right internal/external capsule. In the tract-level analyses, left egocentric, left allocentric, and right egocentric neglect were each associated with damage to the inferior fronto-occipital fasciculus and inferior longitudinal fasciculus, though the strength of this relationship varied widely across the considered visuospatial neglect subtypes. In network-level analyses, left egocentric neglect was significantly associated with damage to a large number of network edges spanning all included right-hemisphere functional networks. Left allocentric neglect was associated with a comparatively restricted subset of right-hemisphere network edges, which did not entirely overlap with the network associated with egocentric neglect. Notably, right egocentric neglect was associated with network edges connecting left- and right-hemisphere areas. Only minimal overlap between the correlates of right and left-lateralised visuospatial neglect was identified. Taken together, these results provide new insights into the neural correlates of visuospatial neglect, a common and debilitating post-stroke neuropsychological impairment (Moore et al. 2021b; Nys et al. 2006; Parton et al. 2004).

### Voxel-level analyses align with previous studies

The results of the voxel-wise lesion-mapping analyses are generally consistent with previous literature. Behavioural scores for egocentric and allocentric neglect were significantly correlated. This result is consistent with past studies, which have concluded that this association is likely due to the fact that egocentric and allocentric neglect arise from damage to independent but spatially proximal brain regions, and that these are therefore more likely to be affected by the same lesion (Chechlacz et al. 2010, 2012b; Kenzie et al. 2015; Moore et al. 2021a). Past behavioural work has provided strong evidence that egocentric and allocentric neglect are doubly dissociated impairments which are separable in terms of their neural correlates, lateralisation of occurrence, behavioural phenotypes, and associated recovery trajectories (Chechlacz et al. 2012a, b; Demeyere and Gillebert 2019; Turgut et al. 2017). In terms of anatomy, left allocentric and egocentric neglect have been associated with independent but partially overlapping voxel clusters centred within the right temporo-parietal cortex (Chechlacz et al. 2010; Kenzie et al. 2015; Moore et al. 2021a, b). In our analyses, although many ROIs were impacted in both allocentric and egocentric neglect, the voxels within each ROI did not always overlap. The voxel clusters associated with left egocentric neglect extended more anteriorly than those associated with left allocentric neglect, and left allocentric neglect was associated with lesions in the lateral occipital cortex and neighbouring occipital fusiform gyrus regions, which were not associated with left egocentric neglect impairment.

Right egocentric neglect was associated with damage to the left-hemisphere occipital fusiform gyrus, inferior frontal gyrus, and anterior limb of the internal capsule. Previous literature has linked right egocentric neglect to damage within occipital cortex, including the occipital fusiform cortex, lingual gyrus, and intercalcarine cortex (Moore et al. 2021a, b). Other work has suggested an association between right egocentric neglect and damage to left anterior fronto-temporal regions, including the frontal operculum and anterior temporal lobe (Beume et al. 2017; Suchan & Karnath 2011). In our study, voxels within both these previously identified but spatially distinct regions were associated with right egocentric neglect. Only one previous study identified voxels significantly associated with right allocentric neglect, and that work implicated the white matter of the left internal capsule (Moore et al. 2021a, b). We replicated this result in the current study, and also identified significant ipsilateral correlates of right allocentric neglect.

Specifically, here, we show that right allocentric neglect is associated with ipsilateral damage to voxels in the anterior medial basal ganglia of the right hemisphere. This finding was not entirely unexpected as ipsilesional visuospatial neglect deficits have been documented in several previous single case and group-level studies (Kim et al. 1999; Kwon & Heilman 1991) and 19/53(33.9%) of included right allocentric patients exhibited right-hemisphere lesions. Some past work has suggested that ipsilesional visuospatial neglect following right-hemisphere lesions represents a compensatory mechanism adopted by patients who initially exhibit left visuospatial neglect in the very early stages following stroke (Kwon & Heilman 1991). However, several studies have identified patients with right ipsilesional visuospatial neglect with no evidence of having first demonstrated left visuospatial neglect, suggesting that this conceptualisation cannot account for all cases of ipsilesional visuospatial neglect (Kim et al. 1999; Sacchetti et al. 2015). Instead, right ipsilesional visuospatial neglect might in some instances be caused by damage to right-hemisphere systems responsible for distributing attention across both the right and left visual fields (Heilman and Abell 1980). This conceptualisation is supported by the findings of the current study, as our included patients demonstrated ipsilesional visuospatial neglect in the acute phase, before compensatory mechanisms would be likely to have exerted their effects (Kwon and Heilman 1991). Additionally, bilateral inferior frontal and temporal regions are key anatomical components of the ventral visual processing stream, and are involved in object-level perceptual and recognition processes (Borowsky et al. 2007; Grill-Spector 2003; Quiroga et al. 2005). Previous research has indicated that the internal capsule contains a number of fibres which diverge to connect parietal, temporal, occipital, and sensory–motor cortices (Zarei et al. 2007). Damage to these connections may disrupt communication within fronto-temporal networks, and may therefore be related to the occurrence of allocentric neglect impairment.

The analyses presented in this study aimed to determine the correlates of left and right visuospatial neglect symptoms, regardless of the lateralisation of underlying damage. However, it is also possible to quantify the correlates of visuospatial neglect following left and right-hemisphere lesions independently (as done in Supplementary Analysis 1). Within the unilateral right-hemisphere sample, voxel-level analyses identified correlates for left egocentric, left allocentric, and right allocentric neglect which were similar to those reported in the main bi-hemispheric analyses. All other hemisphere-specific analyses yielded very few significant results.

There are several reasons for the relatively small number of significant correlates in the unilateral analyses. Perhaps, most importantly, hemisphere-specific analyses contain lesion samples which are both smaller and less anatomically diverse, thereby reducing voxel-wise statistical power. Within the main analyses (not separated based on lesion side), the smallest impairment subtype (right allocentric neglect) contained 49 individuals. The split-hemisphere analysis impairment groups contained a mean of 36.3 participants, with the smallest group size being 12. The consequent reduction in statistical power, coupled with the conservative nature of our analyses, errs on the side of producing false negatives for the hemisphere-specific analysis. These results, reported in the Supplementary Materials, likely differ from past studies, because we applied strict corrections for multiple comparisons and for lesion volume, and we employed a strict minimum overlap threshold. A recent systematic review by our group found that the majority of previous lesion mapping studies of neglect (24 of 34) did not employ corrections for lesion size (Moore et al. 2023a, b). There is considerable diversity in the results of previous lesion mapping investigations of neglect, with 34 past studies reporting over 90 different ROIs as neural correlates of the neglect syndrome (Moore et al 2023a, b). Moreover, no previously published work has conducted tract- or network-level disconnection analyses of neglect following left-hemisphere lesions (Moore et al. 2023a, b). Considered in this context, there are several reasons why the results of our split-hemisphere analyses do not align with previous studies, including differences in design, statistical power, sample characteristics, and approaches to correcting for multiple comparisons. We have included these analyses in the Supplementary Materials for transparency, but do not believe that they offer sufficient tract- and network-level statistical power to support confident interpretation.

Overall, the results of this study suggest that right allocentric neglect is an anatomically diverse condition which can result from either right or left-hemisphere damage. This finding aligns well with the theory that disruptions to dominant right-hemisphere attentional networks can cause ipsilesional attentional deficits in some cases (Kim et al. 1999; Kwon & Heilman 1991).

### Neglect is associated with tract-level disconnection

Our tract-level results largely agree with those reported previously. Left egocentric neglect was associated with damage to 21/45 included right-hemisphere white matter tracts including the corticothalamic pathway, U-fibres, inferior fronto-occipital fasciculus, and superior longitudinal fasciculus. Previous studies have suggested that egocentric neglect is associated disruption of the superior longitudinal fasciculus (Chechlacz et al. 2012a). Damage to this tract is the most commonly reported correlate of visuospatial neglect, with 17/34 existing lesion-mapping studies identifying it as a correlate of neglect (Moore et al. 2023b). Other studies have identified the inferior fronto-occipital fasciculus (Toba et al. 2017), the splenium (Lunven et al. 2013), the inferior longitudinal fasciculus (Machner et al. 2018), and the arcuate fasciculus (Machner et al. 2018) as being associated with egocentric neglect impairment.

Conversely, left allocentric neglect was associated with disconnection of right-hemisphere tracts including the U-fibres and superior/middle longitudinal fasciculus. Notably, all tracts associated with left allocentric impairment were also significantly predictive of left egocentric impairment. A large voxel-wise lesion-mapping analysis conducted by Chechlacz et al. (2010) concluded that egocentric and allocentric neglect were associated with distinct and overlapping cortical correlates, but shared only common white matter correlates including the superior longitudinal, inferior fronto-occipital, and inferior longitudinal fasciculi. Right allocentric neglect was not associated with any significant tract-level predictors even after less conservative FDR corrections were applied. This null result is likely due to a lack of tract-level statistical power stemming from the comparatively diverse locations of lesions in patients exhibiting right allocentric neglect. For example, of the 53 patients exhibiting right allocentric neglect, the most commonly impacted tract was the right external capsule (*n* = 9 patients). Thus, despite the very large overall sample size and good lesion coverage, our right allocentric tract-level analyses were comparatively underpowered.

In tract-level analyses, right egocentric neglect was mainly associated with disconnection within anatomical homologues of tracts reliably associated with left egocentric neglect impairment. Specifically, only damage to the cingulum was associated with right but not left egocentric neglect impairment. Previous investigations characterising white matter damage implicated in right visuospatial neglect reported similar findings. For example, Toba et al. (2022) characterised patterns of white matter damage in three left-hemisphere patients with right egocentric neglect. They concluded that damage to the superior longitudinal fasciculus is associated with right visuospatial neglect impairment, and that the white matter anatomy of right egocentric neglect largely mirrors the white matter correlates of left egocentric neglect. However, large white matter tracts are not functionally homogeneous (Nakajima et al. 2020). Instead, different subdivisions of these tracts selectively support a range of cognitive functions by facilitating communication between a diverse range of cortical areas. Considering tract disconnection as a whole, rather than accounting for functionally distinct subdivisions, may mask critical variance in the patterns of disconnection associated with different visuospatial neglect impairments. Network-based lesion-mapping approaches provide a solution to this problem by considering more fine-grained, pair-wise rather than tract-level disconnection statistics.

### Neglect subtypes are associated with distinct patterns of network-level disconnection

Our network-level lesion-mapping revealed that left egocentric neglect is associated with many disconnected network edges spanning the majority of the right hemisphere. Previous investigations in smaller samples of visuospatial neglect patients have identified similarly large and distributed networks associated with left egocentric neglect (Saxena et al. 2022). Notably, the methods used in this study are designed to be highly conservative and to prioritise specificity over sensitivity, yet still yielded a large significant network. This finding is important as it strongly suggests that left egocentric neglect is not caused by damage to any single critical lesion site but can instead be caused by damage at many different sites across a diffuse right-hemisphere attentional network (Bartolomeo et al. 2007). Our findings align well with previous disconnection and network-based analyses of visuospatial neglect (Lunven et al. 2013; Thiebaut de Schotten et al. 2014; Toba et al. 2017), and help account for the wide range of correlates of left egocentric neglect reported in previous univariate lesion-mapping studies 7/09/2023 9:37:00 PM.

Ours is the first study to identify statistically significant network-level correlates of allocentric neglect deficits. Like left egocentric neglect, left allocentric neglect was associated with a disconnection within network edges across the right hemisphere. These affected edges largely overlapped with those associated with left egocentric neglect, but three edges were associated with allocentric, but not egocentric neglect. Interestingly, these edges included elements of the dorsal-attention network, as well as other connections traditionally associated with the voluntary control of visuospatial attention (Corbetta and Shulman 2011). Specifically, these edges implicated in allocentric, but not egocentric, neglect were a connection between the frontal eye fields dorsal-attention network division and the lateral prefrontal cortex, an edge connecting the default network dorsal/medial prefrontal cortex and the posterior dorsal attentional network, and an edge connecting the somatic-motor network division 6 and 7.

It has been suggested that egocentric neglect may be caused by damage to dorsal attentional areas, whereas allocentric neglect may be more closely related to ventral attentional network lesions (Hillis et al. 2005; Medina et al. 2008). This suggestion is not supported by our findings. We found that left egocentric and left allocentric neglect were associated with damage to connections within both the dorsal and ventral attention networks. Although there was a high co-occurrence of egocentric and allocentric neglect in our sample, this association alone cannot explain why some dorsal-attention structures were associated with allocentric but not egocentric neglect. Overall, the findings of our network-level analyses imply that the anatomical division between allocentric and egocentric neglect may be more nuanced than a simple division between dorsal/ventral attention networks.

Past research has suggested that the dorsal and ventral attention networks do not work in isolation, but instead dynamically interact to direct attention to relevant stimuli (Vossel et al. 2014). The superior longitudinal fasciculus is thought to play a key role in facilitating this interaction, as the second branch of this tract connects the prefrontal component of the dorsal network with the parietal division of the ventral attention network (Nakajima et al. 2020; Thiebaut de Schotten et al. 2014; Wang et al. 2016). This structure provides an anatomically plausible framework for facilitating communication between these networks and is also the region which has been most consistently related to visuospatial neglect impairment (Chechlacz et al. 2012a; Moore et al. 2023a, b). Critically, damage to the superior longitudinal fasciculus has been associated with both egocentric and allocentric neglect both in the current study and by past research (Chechlacz et al. 2010, 2012b). It therefore seems plausible that different types of visuospatial neglect may be related to different impairments of information transfer between dorsal and ventral attention networks rather than to a selective deficit to one of these networks alone (Vossel et al. 2014).

Conversely, visuospatial neglect may result from a deficit which is common across both dorsal and ventral attention networks. For example, effective visual attention guides processing toward behaviourally relevant stimuli, but the categorisation of stimuli into behaviourally relevant or irrelevant can be handled independently of visual attention (Bar et al. 2006; Bourgeois et al. 2018; Summerfield and Egner 2009). Prefrontal executive mechanisms are responsible for guiding goal-directed behaviour and influence the distribution of both egocentric and allocentric spatial attention (Karnath et al. 2002). It is possible that visuospatial neglect arises from lateralised imbalances in how the behavioural relevance of stimuli is computed or how this information is communicated to the dorsal/ventral systems which then direct attention. This possibility aligns with past work documenting abnormalities in initiation in patients with visuospatial neglect (Antoniello and Khazaei 2019; Kwon and Heilman 1991; Mattingley et al. 1998) and with past work suggesting that visuospatial neglect may be related to abnormalities in integration between goal-directed and stimulus-related information (Bays et al. 2010). Overall, additional research will be needed to better characterise the computational and cognitive impairments underlying egocentric and allocentric neglect. Specifically, future investigations should aim to identify the core cognitive impairments involved in different visuospatial neglect symptoms to more precisely quantify differences between egocentric and allocentric neglect and to further fundamental understanding of these common and debilitating neuropsychological impairments.

Our investigation is also the first to identify network-level correlates of right egocentric neglect. Strikingly, right egocentric neglect impairment was associated with network edges primarily linking the right- and left-hemisphere dorsal attention, ventral attention, default, and somatic-motor networks. Notably, these results did not survive highly conservative Bonferroni corrections for multiple comparisons but were significant when standard, less conservative FDR corrections were applied (Mirman et al. 2018). The identified network-level correlates of right egocentric neglect align with previously documented cases of right egocentric neglect after both right- and left-hemisphere lesions (Kim et al. 1999; Moore et al. 2021a, b), but do not clearly agree with past voxel-wise lesion-mapping studies of right egocentric neglect. Previous voxel-wise lesion-mapping analyses linked right egocentric neglect impairment with damage to the left-hemisphere anterior/middle temporal lobe and insular/opercular region, as well as the occipital fusiform, but they did not identify any significant ipsilesional correlates of right egocentric neglect (Beume et al. 2017; Moore et al. 2021a; Suchan and Karnath 2011). However, several studies that linked right egocentric neglect to exclusively left-hemisphere regions excluded patients with right-hemisphere damage, precluding the detection of similar bilateral disconnection relationships (Chechlacz et al. 2012a). Our findings strongly suggest that right egocentric neglect can be caused by a range of both right- and left-hemisphere lesions which may impact these critical network connections.

There has been considerable debate as to whether right visuospatial neglect is caused by damage to left-hemisphere homologues of the right-hemisphere areas associated with left visuospatial neglect. Previous literature has concluded that the left-hemisphere correlates of right visuospatial neglect mirror (or partially mirror) right-hemisphere areas associated with left visuospatial neglect (Beume et al. 2017; Suchan and Karnath 2011; Toba et al. 2022). Our findings suggest that this “partial mirroring” may be a consequence of the diversity of right-hemisphere lesions which cause visuospatial neglect, rather than indicating a true underlying anatomical similarity (Moore et al. 2023a, b). Previous studies suggesting a common anatomy of left and right visuospatial neglect have considered evidence exclusively from a single hemisphere, and have drawn conclusions based on comparing these findings with the existing literature (Beume et al. 2017; Suchan and Karnath 2011; Toba et al. 2022). Here, we undertook a direct comparison of lesions across hemispheres in a representative sample of stroke survivors. Our findings suggest that there are few similarities in the anatomy of left- and right-hemisphere egocentric neglect. Specifically, in voxel-wise lesion-mapping, only 32 (0.04%) of the voxels associated with left egocentric neglect overlapped with voxels in the opposite hemisphere associated with right egocentric neglect. Similarly, for left and right allocentric neglect, there were no shared voxels between homologous regions of the left and right hemispheres. Our tract-based analyses revealed substantial overlap between left and right egocentric neglect, but considering tract disconnection as a whole rather than accounting for functionally distinct subdivisions may mask critical variance in the patterns of disconnection associated with different visuospatial neglect impairments. For this reason, the results of network-level lesion-mapping are more informative for identifying similarities and differences between right and left visuospatial neglect. In network-level analyses, right and left egocentric neglect involved qualitatively different connectivity patterns with no overlap between homologous network edges involved in left and right visuospatial neglect.

### Right and left neglect are not anatomically homologous

These results suggest that right and left egocentric neglect are not anatomically homologous, but instead may be caused by damage to a shared but hemispherically asymmetric attention network. In other words, the anatomy of right-lateralised visuospatial neglect does not appear to be a “mirror image” of the anatomy implicated in left-lateralised visuospatial neglect. Instead, right-lateralised visuospatial neglect may involve damage to the same bilateral, hemispherically asymmetric network underlying left-lateralised visuospatial neglect, confirming the right-hemisphere dominance for spatial attention (Corbetta and Shulman 2011; Heilman and Abell 1980; Weintraub and Mesulam 1987). This finding aligns with previous research documenting a comparatively lower incidence and severity of right versus left egocentric neglect (Stone et al. 1993; Ten Brink et al. 2017). For example, left-hemisphere lesions may be less likely to cause visuospatial neglect than right hemisphere lesions due the substantially lower probability that these lesions will intersect with critical attention network streamlines. The reduced severity of right-lateralised visuospatial neglect deficits may also be linked to the relatively lower proportion of network edges which would be expected to be disrupted by a left-hemisphere versus a right-hemisphere stroke lesion (Stone et al. 1993; Ten Brink et al. 2017).

## Limitations

We employed routinely collected behavioural data and clinical neuroimaging. This approach allows the inclusion of a large and representative sample but does introduce some potential limitations. Ischemic stroke lesions are subject to temporal development and are not always well defined on acute CT scans (González 2005; Merino and Warach 2010). This issue is inherent in all lesion-mapping methodologies: false-positive and false-negative delineation errors are often made on higher quality (e.g., MR) scans as well (Moore et al. 2023a, b). However, we have shown that CT-derived lesion masks perform with similar accuracy to MR-derived masks (Moore et al. 2023a, b) and that CT-derived lesion masks can reliably uncover anatomical patterns of functional specialisation in stroke (Moore and Demeyere 2022). For these reasons, any noise arising from the use of routinely collected clinical scans is not likely to have systematically biased the outcomes, especially in light of the very large sample size employed here. Similarly, our analyses relied on normatively defined white matter tracts to generate both the tract-level and network-level disconnection statistics. This approach is less accurate than employing diffusion imaging to map white matter tracts in each included patient and may introduce some noise into the considered anatomical data. However, diffusion imaging was not available for this very large patient sample. Similarly, detailed visual field assessments were not available for the included sample meaning that we were unable to control for the presence/absence of visual field deficits. However, past research has demonstrated that allocentric and egocentric neglect can be dissociated from visual field loss, and that the OCS cancellation task can assess visuospatial neglect independently of early visual field deficits (Bickerton et al. 2011; Demeyere et al. 2015).

Visuospatial neglect is most reliably detected and quantified by comparing performance across multiple standardised behavioural tests (Halligan et al. 1989; Huygelier et al. 2020; Moore et al. 2022). In this retrospective analysis, data from only one visuospatial neglect test were available. Though additional tasks may have improved sensitivity to visuospatial neglect, it was not feasible to include these given the acute acquisition and presence of relatively severe visuospatial neglect impairments in many patients. Notably, the OCS Cancellation Task has been shown to be highly sensitive relative to other standard tasks and is recommended as the best method to detect visuospatial neglect impairment in cases where it is only possible to use one test (Moore et al. 2022). This study classified the occurrence of egocentric and allocentric neglect based on the standard OCS scoring criteria. It is possible that using different scoring thresholds would yield different results, but the use of these alternative metrics is not supported by normative data. This study employed a large and representative sample of stroke survivors (*n* = 480), but not all conducted analyses had equal statistical power. Specifically, right allocentric neglect was present in a comparatively small number of patients (*n* = 49) who exhibited greater diversity in lesion anatomy. This diversity implies that individual voxel-, tract-, and network-level analyses aiming to investigate right allocentric neglect may be underpowered, but also provides important and novel insight into the anatomy of this condition. Overall, the results of this study suggest that right allocentric neglect is an anatomically diverse condition which can result from either right- or left-hemisphere damage. Future studies should aim to explore this diversity and quantify more fine-grained anatomical patterns among patients with right allocentric neglect.

Lesion-mapping approaches also have their limitations, as has been highlighted in the previous published work (Mah et al. 2014; Yourganov et al. 2016). Voxel-wise approaches cannot accurately characterise the anatomy of disconnection syndromes and are subject to spatial bias due to non-random distributions in lesion locations (Bates et al. 2003; Mah et al. 2014). Tract-level analyses assume tract-level functional homogeneity and risk masking critical variance in lesion anatomy (Nakajima et al. 2020). Network-level analyses rely on group-level anatomical templates and may not sufficiently account for variance in functional and structural connectivity between individuals (Saxena et al. 2022; Yeh et al. 2018). For these reasons, consensus outcomes across multiple levels of lesion-mapping analyses, as we have provided here, provide stronger evidence than any single methodology in isolation. Multivariate lesion-symptom mapping techniques may provide an effective control for many of the statistical limitations within traditional univariate approaches (Pustina et al. 2018; Yourganov et al. 2016; Zhang et al. 2014). In addition to impacting different frames of reference, visuospatial neglect patients often display a variety of behavioural phenotypes, impacted sensory modalities, and functional outcome (Guilbert et al. 2016; Laplane and Degos 1983; Moore and Demeyere 2017; Ten Brink et al. 2019). Future research is needed to quantify the anatomy of each of these distinct subtypes and to link neuroanatomical patterns to specific functional outcomes.

As the goal of the current study was to explore the correlates of right and left visuospatial neglect (regardless of the hemisphere lesioned), we do not make any claims as to the correlates of “contralesional” versus “ipsilesional” behavioural visuospatial neglect. In this study, we compared the correlates of visuospatial neglect deficits across hemispheres by quantifying the degree of overlap in significantly associated correlates across hemispheres. This analysis represents an overlap comparison rather than a direct statistical comparison. Future research could extend this approach by conducting direct statistical comparisons between the correlates associated with egocentric and allocentric neglect.

## Conclusion

The findings of this study elucidate the voxel-wise, tract-level, and network-level correlates of egocentric and allocentric neglect. Overall, this investigation’s results strongly suggest that right and left egocentric neglect are not anatomically homologous but involve damage to a shared, hemispherically asymmetric attention network. This implication is critically important to consider in the context of clinical practice as it suggests that all stroke patients, regardless of lesion location, should be screened for visuospatial neglect (Moore et al. 2019). Our study also has important theoretical implications as it highlights the complexity of the networks responsible for accurately distributing attention over space, and identifies novel hemispheric asymmetries within this network (Beume et al. 2017; Suchan and Karnath 2011; Toba et al. 2022). Cumulatively, these findings provide novel insight into the neural correlates of spatial attention.

### Supplementary Information

Below is the link to the electronic supplementary material.

Supplementary file1 (DOCX 7631 KB)

## Data Availability

The data and analysis code associated with this project have been made openly available on the Open Science Framework (https://osf.io/qwd8k/), except for patient lesion masks and clinical brain scans which are available upon request.
