# Supplementary material for: Comprehensive voxel-wise, tract-based, and network lesion mapping reveals unique architectures of right and left visuospatial neglect
Source: Brain Struct Funct. 2023 Sep 11;228(9):2067–87. doi: 10.1007/s00429-023-02702-2 (PMC10587018; doi:10.1007/s00429-023-02702-2)
Supplement: Supplementary file 1 — Supplementary file1 (DOCX 7631 KB) [file 429_2023_2702_MOESM1_ESM.docx]

**Supplementary Materials:**

**
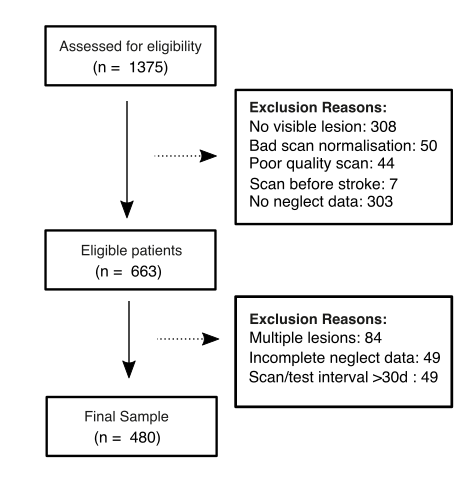
**

***Supplementary Figure 1:*** *Patient inclusion and exclusion numbers and reasons. D = days.*

*
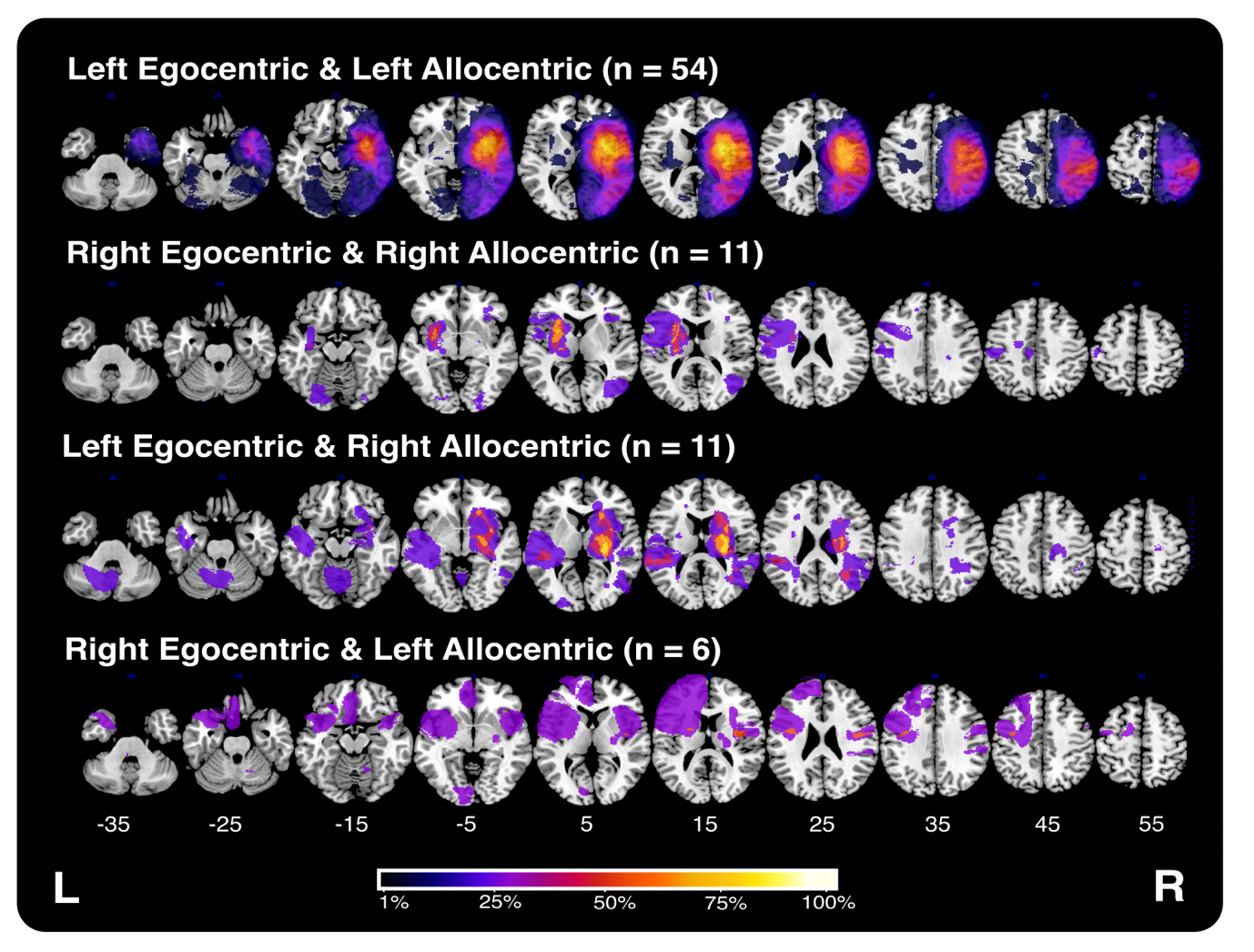
*

***Supplementary Figure 2:*** *Grouped lesion overlays for the 82 patients exhibiting both egocentric and allocentric neglect deficits. As each overlay represents data from a variable number of patients, the colour scale depicts the percentage of included individuals with damage in each voxel relative to the maximum overlap within each subgroup.*

***
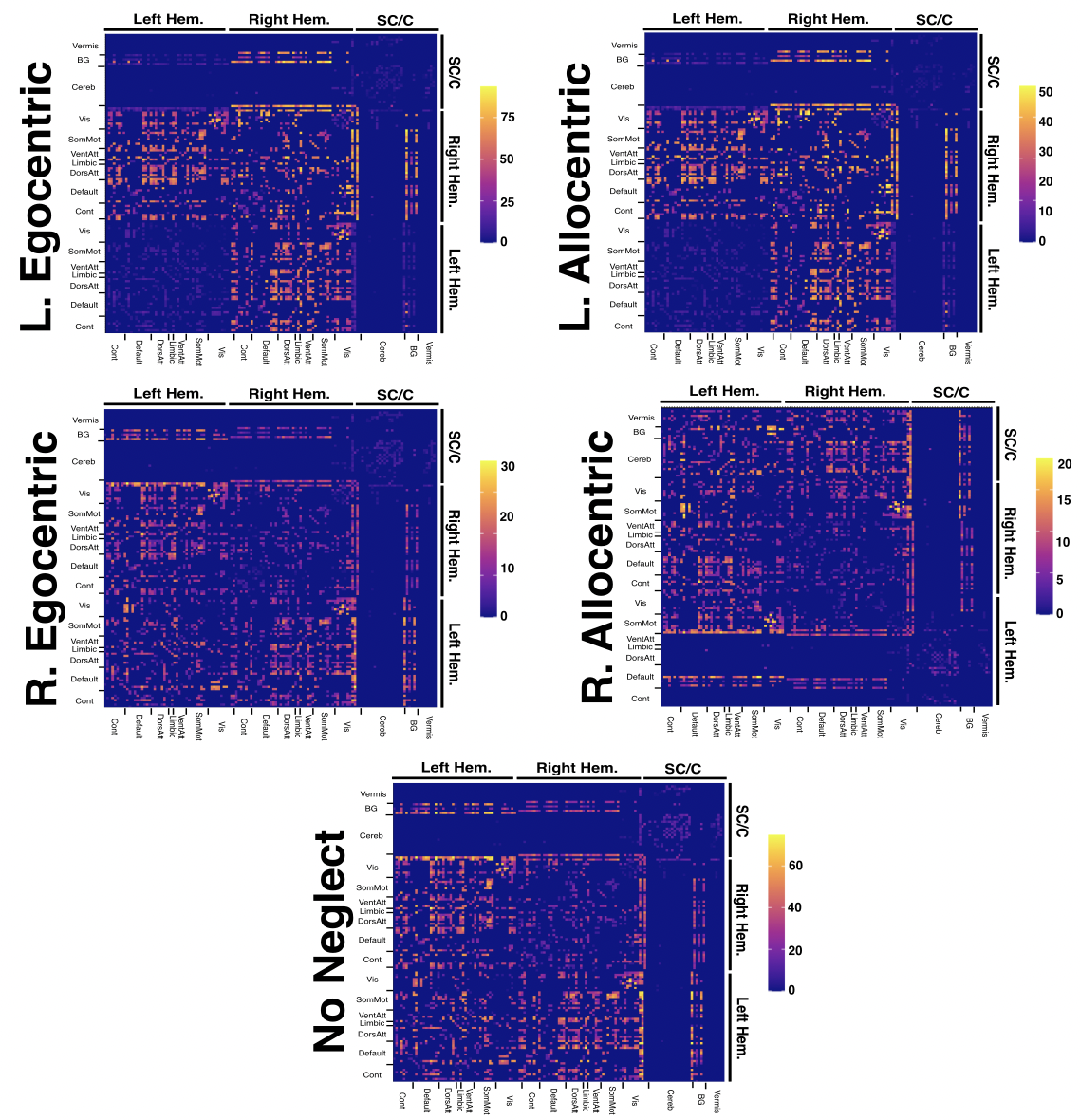
 Supplementary Figure 3:*** *Disconnection matrices showing the number for each patient subgroup with at least 50% disconnection at each considered network edge. Cell colour represents number of patients. X and Y atlases are arranged according to the parcels and network subdivisions reported in the Schaefer-Yeo Atlas (7 networks, 100 nodes). SC/C = subcortical / cerebellar , Cereb = cerebellar, BG = basal ganglia, Vis = visual network, SomMot = somatic motor network, VentAtt = ventral attention network, Limbic = limbic network, DorsAtt = dorsal attention network, Default = default network, Cont = control network.*

*Supplementary Left and Right Hemisphere Subgroup Lesion Mapping Analyses:*

**
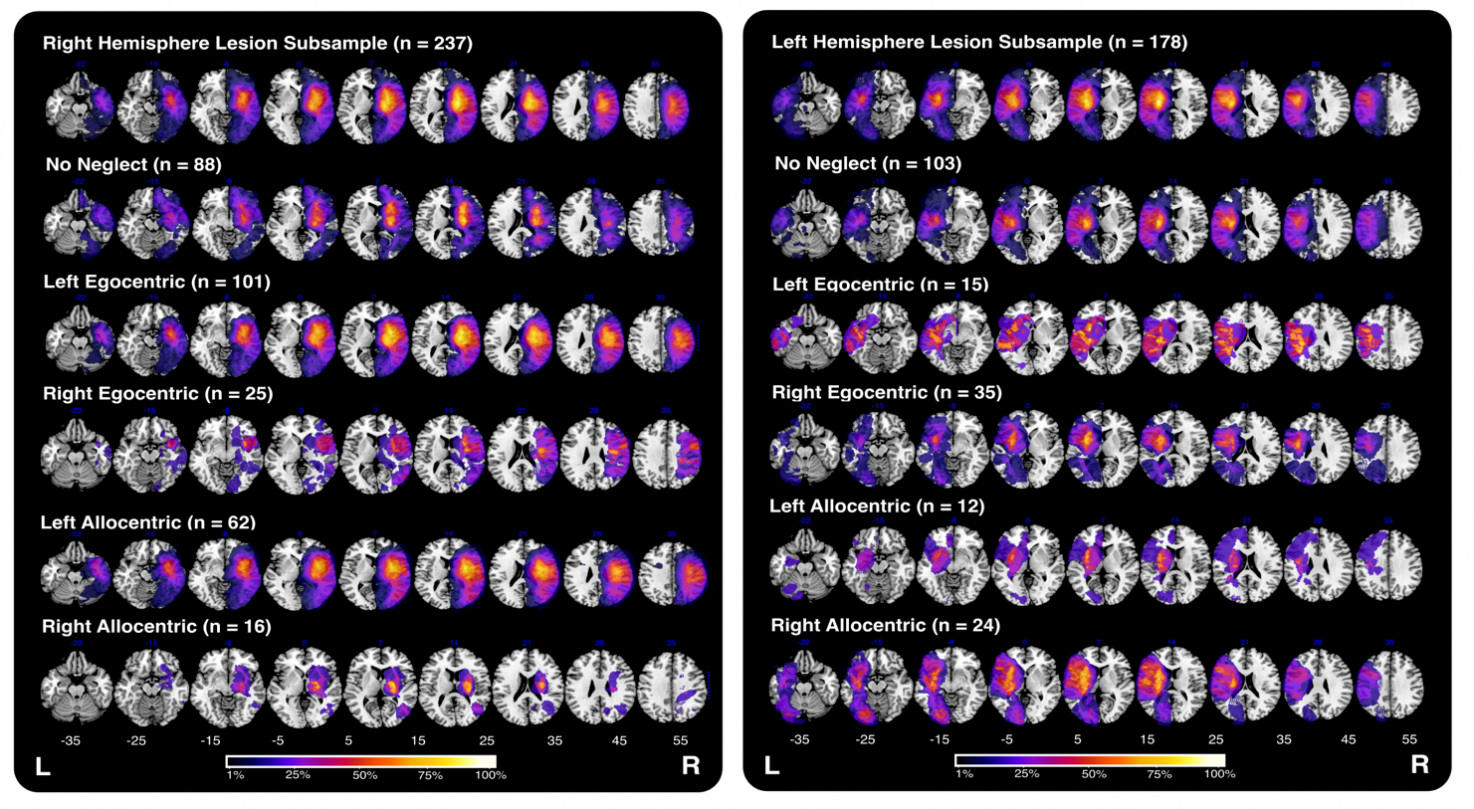
**

*Figure 4: Lesion overlays of the right- and left-hemisphere lesion samples grouped according to the category of neglect impairment.* *As each overlay represents data from a variable number of patients, the colour scale depicts the percentage of included individuals with damage in each voxel relative to the maximum overlap within each subgroup.*


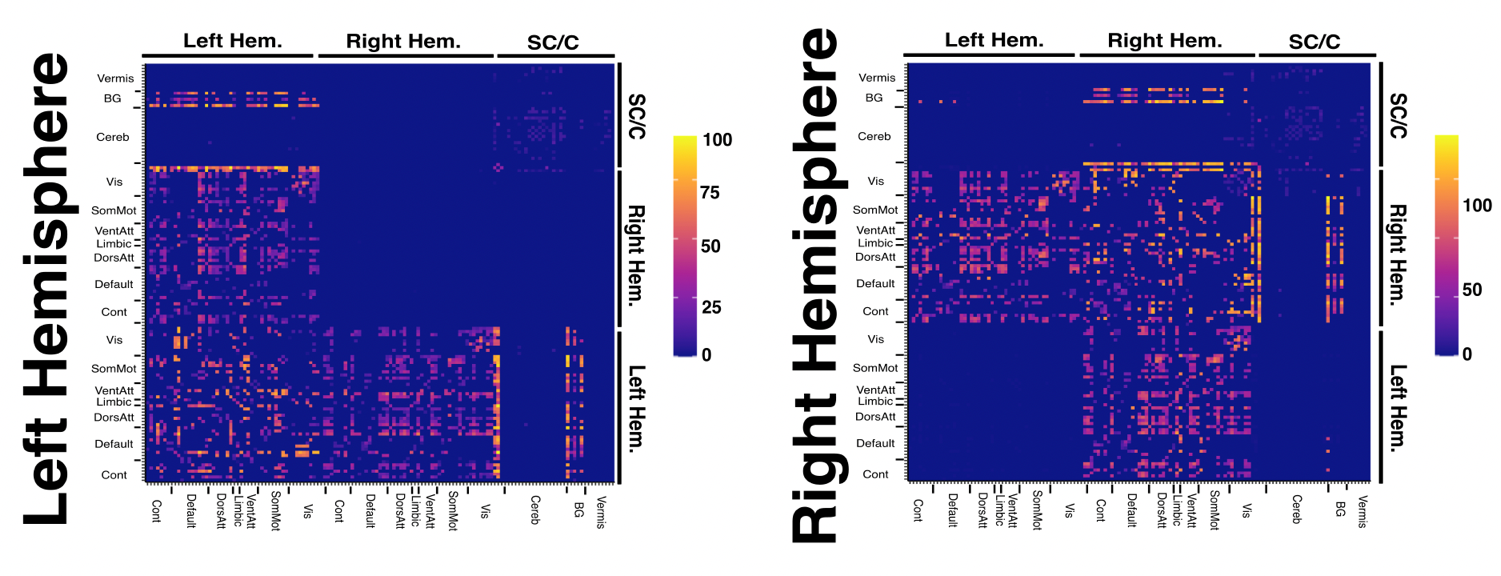


**Figure 5:** *Disconnection matrices illustrating the number of included patients with at least 50% disconnection at each considered network edge for the left and right hemisphere lesion subsamples. Cell colour represents number of patients. X and Y atlases are arranged according to the parcels and network subdivisions reported in the Schaefer-Yeo Atlas (7 networks, 100 nodes). SC/C = subcortical / Cerebellar, Cereb = cerebellar, BG = basal ganglia, Vis = visual network, SomMot = somatic motor network, VentAtt = ventral attention network, Limbic = limbic network, DorsAtt = dorsal attention network, Default = default network, Cont = control network.*

*Voxel-wise Lesion Mapping Results:*

Within the right hemisphere sub-sample (Bonferroni-corrected alpha= 1.51x =10^-7^), left egocentric neglect was significantly associated with 1327 voxels located within the parietal operculum and posterior division of the supramarginal gyrus (Supplementary Figure 6). Left allocentric neglect was associated with 3555 voxels, largely centred within the anterior division of the supramarginal gyrus. Right egocentric neglect was associated with 54 significant voxels located in the temporo-occipital fusiform cortex. Finally, right allocentric neglect was associated with 1293 voxels centred within the internal capsule.

Within the left hemisphere subsample, no voxels were reliably associated with left egocentric, left allocentric, or right egocentric neglect (FDR corrected). Right allocentric neglect was associated with 90 voxels centred within the anterior limb of the left external capsule (Supplementary Figure 7) (Bonferroni-corrected alpha = 3.6x10^-7^).

**
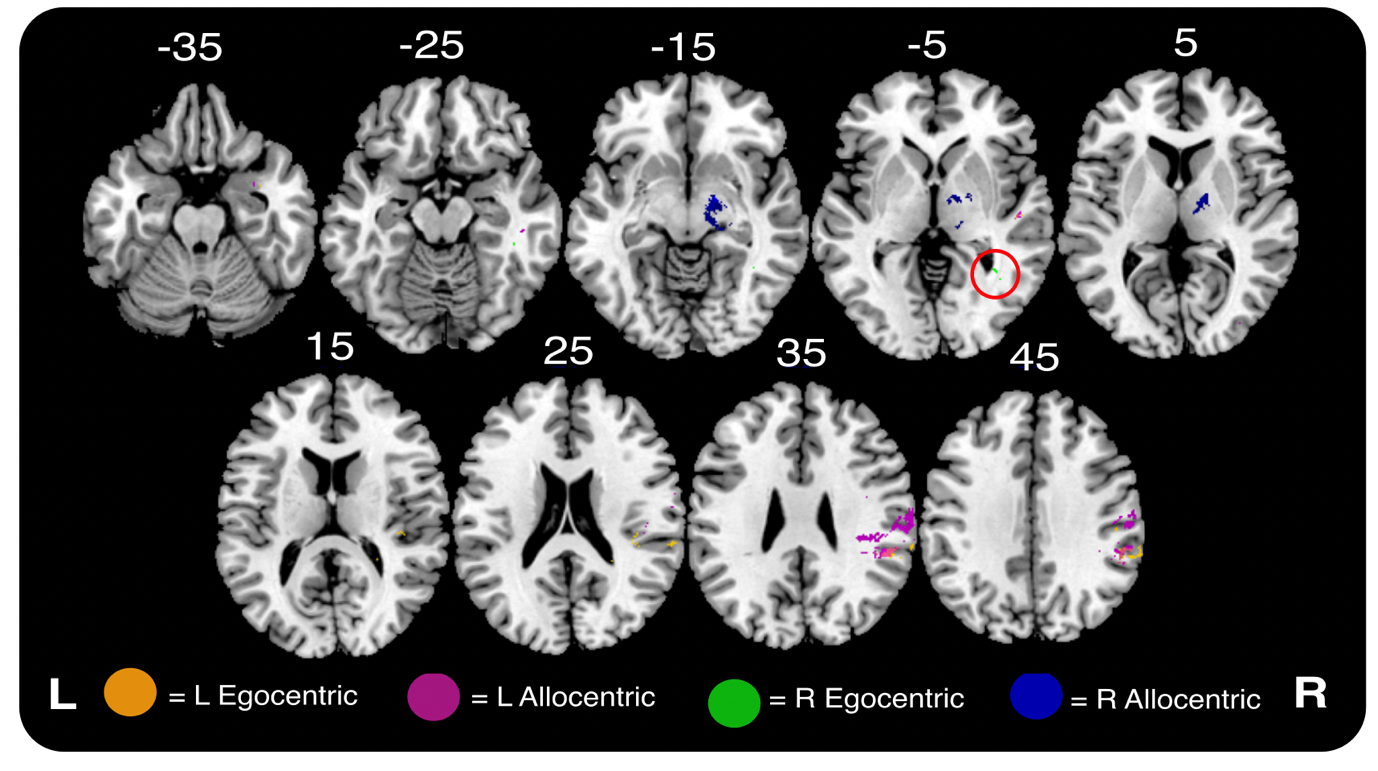
**

***Supplementary Figure 6:*** *Visualisation of significant voxels identified in univariate lesion-mapping analyses of left and right egocentric and allocentric neglect within the right hemisphere sample. All visualised voxels survived highly conservative Bonferroni corrections for multiple comparisons. The significant correlates associated with right egocentric neglect are highlighted by the red circle. MNI z coordinates -35 – 45 are visualised.*

*
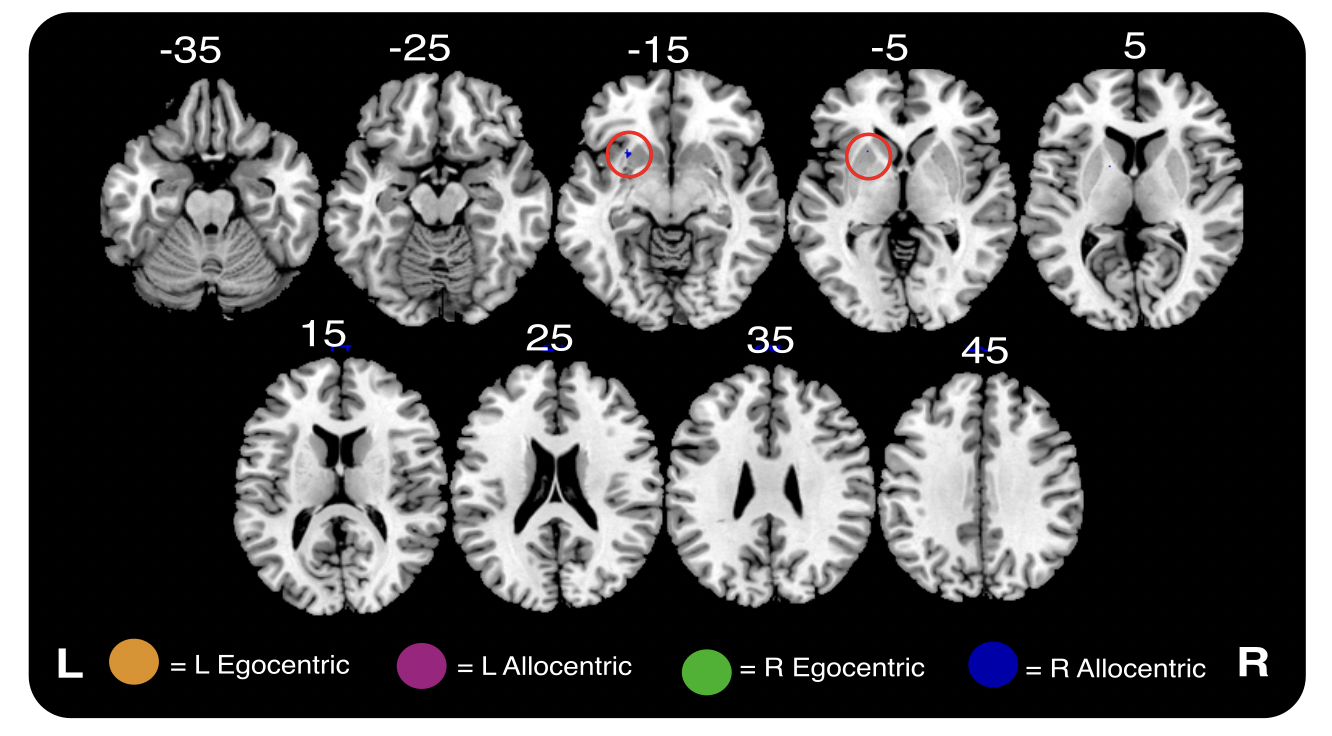
*

***Supplementary Figure 7:*** *Visualisation of significant voxels identified in univariate lesion-mapping analyses of left and right egocentric and allocentric neglect within the left hemisphere sample. All visualised voxels survived highly conservative Bonferroni corrections for multiple comparisons. Only correlates of right allocentric neglect were statistically significant (highlighted in red). MNI z coordinates -35 – 45 are visualised.*

*Tract-level Lesion Mapping:*

Within the right hemisphere subsample, left egocentric neglect was significantly associated with disconnection within the right cingulum (adjusted R^2^ = 0.058) and the dorsal longitudinal fasciculus (adjusted R^2^ = 0.061) (Bonferroni corrected, alpha = 0.0014). No tracts were significantly associated with left allocentric, right egocentric, or right allocentric neglect within the right hemisphere subsample (FDR corrected). Within the left hemisphere subsample, no tracts were significantly associated with left egocentric, left allocentric, right egocentric, or right allocentric neglect (FDR corrected).

*Network-Level Lesion Mapping:*

In the left hemisphere sample, network-level lesion symptom mapping analysis yielded no significant disconnection correlates for left allocentric, right egocentric, or right allocentric neglect (FDR corrected). Left egocentric neglect impairment was significantly associated with damage to one edge connecting the left ventral attention network (medial division 1) with the left lenticular nucleus (Bonferroni corrected). In the right hemisphere sample, no significant disconnection correlates of left egocentric, left allocentric, right egocentric, or right allocentric neglect were identified (FDR corrected).
